# Chinese expert consensus on the diagnosis and treatment of coronary microvascular diseases (2023 Edition)

**DOI:** 10.1002/mco2.438

**Published:** 2023-12-19

**Authors:** Wenqiang Chen, Mei Ni, He Huang, Hongliang Cong, Xianghua Fu, Wei Gao, Yuejin Yang, Mengyue Yu, Xiantao Song, Meilin Liu, Zuyi Yuan, Bo Zhang, Zhaohui Wang, Yan Wang, Yundai Chen, Cheng Zhang, Yun Zhang

**Affiliations:** ^1^ The National Key Laboratory for Innovation and Transformation of Luobing Theory The Key Laboratory of Cardiovascular Remodeling and Function Research, Chinese Ministry of Education, Chinese National Health Commission and Chinese Academy of Medical Science Department of Cardiology Qilu Hospital of Shandong University Jinan Shandong China; ^2^ Department of Cardiology Sir Run Run Shaw Hospital affiliated with Zhejiang University School of Medicine Hangzhou China; ^3^ Department of Cardiology Tianjin Chest Hospital, Tianjin University Tianjin China; ^4^ Department of Cardiology The Second Hospital of Hebei Medical University Shijiazhuang Hebei China; ^5^ Department of Cardiology Peking University Third Hospital Beijing China; ^6^ Department of Cardiology Fuwai Hospital, National Center for Cardiovascular Diseases, Chinese Academy of Medical Sciences and Peking Union Medical College Beijing China; ^7^ Department of Cardiology Beijing Anzhen Hospital, Capital Medical University Beijing China; ^8^ Department of Geriatrics Peking University First Hospital Beijing China; ^9^ Department of Cardiology The First Affiliated Hospital of Xian Jiaotong University Xian China; ^10^ Department of Cardiology First Affiliated Hospital, Dalian Medical University Dalian Liaoning China; ^11^ Department of Cardiology Union Hospital, Tongji Medical College, Huazhong University of Science and Technology Wuhan China; ^12^ Department of Cardiology Xiamen Cardiovascular Hospital, Xiamen University Xiamen China; ^13^ Senior Department of Cardiology, Sixth Medical Center of Chinese PLA General Hospital, Beijing, China; for the Basic Research Group, Atherosclerosis and Coronary Heart Disease Group, Interventional Cardiology Group, and Women's Heart Health Group of the Chinese Society of Cardiology

**Keywords:** coronary microvascular disease, myocardial ischemia, myocardial infarction, atherosclerosis, non‐atherosclerotic heart disease, Chinese expert consensus

## Abstract

Since the four working groups of the Chinese Society of Cardiology issued first expert consensus on coronary microvascular diseases (CMVD) in 2017, international consensus documents on CMVD have increased rapidly. Although some of these documents made preliminary recommendations for the diagnosis and treatment of CMVD, they did not provide classification of recommendations and levels of evidence. In order to summarize recent progress in the field of CMVD, standardize the methods and procedures of diagnosis and treatment, and identify the scientific questions for future research, the four working groups of the Chinese Society of Cardiology updated the 2017 version of the Chinese expert consensus on CMVD and adopted a series of measures to ensure the quality of this document. The current consensus has raised a new classification of CMVD, summarized new epidemiological findings for different types of CMVD, analyzed key pathological and molecular mechanisms, evaluated classical and novel diagnostic technologies, recommended diagnostic pathways and criteria, and therapeutic strategies and medications, for patients with CMVD. In view of the current progress and knowledge gaps of CMVD, future directions were proposed. It is hoped that this expert consensus will further expedite the research progress of CMVD in both basic and clinical scenarios.

## Graphical abstract

New recommendations and opinions released in Chinese expert consensus on the diagnosis and treatment of coronary microvascular diseases—2023, as compared with Chinese expert consensus on the diagnosis and treatment of coronary microvascular diseases issued in 2017.

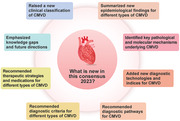

## 1 INTRODUCTION

Since Likoff et al.^1^ first reported the clinical manifestations of a group of patients with a definite diagnosis of coronary heart disease (CHD) but a normal coronary angiogram in 1967, basic and clinical research on coronary microvascular diseases (CMVD) has continued for half a century. In 2013, the European Society of Cardiology (ESC) guidelines for the treatment of stable coronary artery disease (CAD) first recognized CMVD as a clinical type of CHD and made preliminary recommendations for the diagnosis and treatment of CMVD.^2^ In 2017, the Basic Research Group, Interventional Cardiology Group, Women's Heart Health Group, and Atherosclerosis and Coronary Heart Disease Group of the Chinese Society of Cardiology issued “Chinese expert consensus on the diagnosis and treatment of coronary microvascular diseases,” the first expert consensus on CMVD in the literature,^3^ which clarified a number of unclear issues and expedited the progress of basic and clinical research in the field of CMVD in China.

Since 2017, international consensus documents on CMVD have increased rapidly. In 2018, the Coronary Vasomotion Disorders International Study Group (COVADIS) recommended the diagnostic criteria for type 1 coronary artery microvascular dysfunction, namely primary CMVD.^4^ In 2019, the ESC published guidelines for the diagnosis and treatment of chronic coronary syndrome, in which microvascular angina (MVA) pectoris was classified as an important type of chronic coronary syndrome, and corresponding diagnostic and treatment strategies were recommended.^5^ In 2019, the American Heart Association (AHA) issued a scientific statement on the diagnosis and treatment of myocardial infarction (MI) with nonobstructive coronary arteries (MINOCA), stating that MVA pectoris, micro‐vasospasm, and slow coronary flow are important causes of MINOCA.^6^ In 2020, the European Association of Percutaneous Cardiovascular Interventions (EAPCI) and ESC jointly issued a consensus document on ischemia with nonobstructive coronary arteries (INOCA), indicating that CMVD and epicardial coronary artery spasm are the main causes of INOCA.^7^ In 2020, ESC Working Group on Coronary Pathophysiology and Microcirculation published position paper on coronary microvascular dysfunction, dividing CMVD into six clinical types.^8^ These international documents focused on mainly research progress of CMVD, and although some of these documents made preliminary recommendations for the diagnosis and treatment of CMVD, they did not provide classification of recommendations and levels of evidence. Thus, the guidance value of these documents in clinical practice is limited.

In order to summarize recent progress in the field of CMVD, standardize the methods and procedures of diagnosis and treatment of CMVD, and identify the scientific questions for future research, the Basic Research Group, Atherosclerosis and Coronary Heart Disease Group, Interventional Cardiology Group, and Women's Heart Health Group of the Chinese Society of Cardiology held a meeting in November 2021, discussed current issues in the field of CMVD and decided to update the 2017 version of Chinese expert consensus on CMVD. The four working groups adopted the following measures to control the quality of this document: first, a consensus revision committee and a consensus drafting group were formed and their members were nominated by the four working groups; second, search keywords on CMVD in the literature were suggested by the drafting group and approved by the consensus revision committee; third, English and Chinese literatures published from 2016 to 2021 were extensively searched using approved key words and major search engines; fourth, poor quality, repetitive and small sample studies were deleted using the quality standard predefined by the consensus revision committee, and the remaining literatures were classified according to their research area; fifth, a consensus outline was suggested by the drafting group after a thorough investigation of classified literatures, and approved by the consensus revision committee; sixth, the consensus revision committee and drafting expert group held several meetings to discuss the manuscript, which were revised for several times and finally approved by the consensus revision committee and the Chinese Society of Cardiology.

The recommendations of diagnosis and treatment of CMVD in this consensus were based on ACC/AHA criteria as illustrated in Tables 1 and 2.^9^ The following aspects of CMVD were discussed in this consensus sequentially: (1) definition, clinical classification, and epidemiology of CMVD; (2) pathogenetic mechanism of CMVD; (3) diagnostic techniques for CMVD; (4) clinical manifestations and diagnostic criteria of CMVD; (5) treatment of CMVD; and (6) gaps of knowledge and future perspectives. The updated recommendations and opinions in the new consensus were given in Table 3 as compared to the Chinese expert consensus on the diagnosis and treatment of CMVD issued in 2017.

**TABLE 1:** Classes for recommendations.

	Definition
Class I	Treatments or procedures that have been proven and/or unanimously recognized to be beneficial, useful or effective, and recommended
Class II	Treatments or procedures for which there is conflicting evidence and/or a divergence of opinion about the usefulness/efficacy
Class IIa	The evidence/opinion tends to be useful and/or effective, and it is reasonable to apply these treatments or procedures
Class IIb	The relevant evidence/opinions have not been fully proven to be useful and/or valid and can be considered for application
Class III	Treatments or procedures that have been confirmed and/or unanimously recognized as useless and/or ineffective and may be harmful in some cases, and not recommended

**TABLE 2:** Levels of evidence.

	Definition
Level A	Evidence based on multiple randomized clinical trials or meta‐analyses.
Level B	Evidence based on a single randomized clinical trial or multiple nonrandomized controlled trials
Level C	Consensus of expert opinion and/or evidence based on small‐scale studies, retrospective studies, and registration studies

**TABLE 3:** New concepts and recommendations in the consensus 2023 compared with the consensus 2017.

New concepts and recommendations in 2023		
Section 2.2 Clinical classifications		
1	CMVD associated with myocardial ischemia	New
2	CMVD associated with myocardial infarction	
3	CMVD associated with coronary revascularization	
4	CMVD associated with non‐atherosclerotic heart disease	
Section 3 Pathogenetic mechanism of CMVD		
1	Regulatory mechanism of coronary blood flow	New
2	Structural abnormalities of the coronary microcirculation	Updated
3	Microvascular obstruction	New
4	Functional abnormalities of the coronary microcirculation	Updated
Section 4.1 Vasoactive drugs for evaluating coronary microvascular function		
	Regadenoson and nicorandil	New
Section 4.2 Noninvasive techniques for evaluating coronary microvascular function		
1	TTDE	Updated
2	MCE	New
3	SPECT	Updated
4	PET	Updated
5	CMR	Updated
6	CTP	New
Section 4.3 Invasive techniques for evaluating coronary microvascular function		
1	CAG	Updated
2	Bolus and continuous thermodilution	Updated
3	Intracoronary Doppler flow velocity measurement	Updated
4	Diagnostic flow chart	New
Section 5 Clinical manifestations and diagnostic criteria of CMVD		
Diagnostic criteria for different types of CMVD		New
Section 6 Treatment of CMVD		
1	Treatment for CMVD associated with INOCA Risk factor management and lifestyle modifications Coronary and endothelial function test Stratified medical therapy	Updated
2	Treatment of CMVD associated with IOCA Lifestyle modifications Antiatherosclerosis therapy Stratified medical therapy Coronary revascularization	Updated
3	Treatment of CMVD associated with MINOCA Stratified medical therapy Secondary prevention	Updated
4	Treatment of CMVD associated with MIOCA Pharmacological treatment before PCI Pharmacological treatment during PCI Nonpharmacological treatment	Updated
5	Treatment of CMVD after successful intervention for AMI Pharmacological treatment Nonpharmacological treatment	New
6	Treatment of CMVD with non‐atherosclerotic heart disease Treatment of CMVD with myocardial hypertrophy Treatment of CMVD without myocardial hypertrophy	New

Abbreviations: AMI, acute myocardial infarction; CAG, coronary angiography; CTP, computed tomography perfusion; CMR, cardiovascular magnetic resonance; CMVD, coronary microvascular disease; INOCA, ischemia with nonobstructive coronary arteries; IOCA, ischemia with obstructive coronary arteries; MCE, myocardial contrast echocardiography; MINOCA, myocardial infarction with nonobstructive coronary arteries; MIOCA, myocardial infarction with obstructive coronary arteries; PCI, percutaneous coronary intervention; PET, positron emission tomography; SPECT, single‐photon emission computed tomography; TTDE, transthoracic Doppler echocardiography.

## 2 DEFINITION, CLINICAL CLASSIFICATION, AND EPIDEMIOLOGY OF CMVD

### 2.1 Definition

CMVD is a clinical syndrome of acute and chronic myocardial ischemia caused by abnormalities in structure and function of coronary prearterioles, arterioles, and capillaries induced by atherosclerotic and non‐atherosclerotic pathogenic factors.

### 2.2 Clinical classifications

This consensus divides CMVD into four major types and nine sub‐types (Table 4).

**TABLE 4:** Clinical classifications of CMVD.

Classifications
CMVD associated with myocardial ischemia CMVD associated with INOCA CMVD associated with IOCA
2. CMVD associated with myocardial infarction CMVD associated with MINOCA CMVD associated with MIOCA
3. CMVD associated with coronary revascularization CMVD associated with primary PCI CMVD associated with elective PCI CMVD associated with CABG
4. CMVD associated with non‐atherosclerotic heart disease CMVD with myocardial hypertrophy CMVD without myocardial hypertrophy

Abbreviations: IOCA, ischemia with obstructive coronary arteries; CMVD, coronary microvascular disease; INOCA, ischemia with nonobstructive coronary arteries; MIOCA, myocardial infarction with obstructive coronary arteries; MINOCA, myocardial infarction with nonobstructive coronary arteries; PCI, percutaneous coronary intervention; CABG, coronary artery bypass grafting.

### 2.3 Epidemiology

Most epidemiological studies of CMVD were performed in European and American populations, some of which included Asian populations as well. No studies have yet reported ethnic differences in the incidence of CMVD. The incidence of CMVD varies greatly in different studies, ranging from 10% to 80% due to the inconsistency of CMVD definition and diagnostic criteria. Likewise, owing to the differences in endpoints and follow‐up duration, there were significant differences in mortality and the incidence of adverse cardiovascular events in CMVD patients in different studies.^10^ However, among INOCA patients, the incidence of CMVD in females is consistently higher than that in males.

#### 2.3.1 CMVD associated with myocardial ischemia

Available studies showed that women had a higher incidence of CMVD associated with myocardial ischemia. In addition, CMVD increased the incidence of composite endpoints of cardiovascular events as an independent predictor of prognosis.

In a single center study, 329 consecutive patients with angina pectoris underwent coronary angiography (CAG) in whom coronary flow reserve (CFR) after stress testing were measured with myocardial perfusion positron emission tomography (PET) and followed for an average of 3.1 years. Major adverse cardiovascular events (MACE) included cardiovascular death and hospitalization due to heart failure or MI. The extent and severity of angiographically identified coronary disease were estimated using coronary artery disease prognostic index (CADPI) based on the number of stenotic vessels and the severity of coronary artery stenosis (50–100%), with three‐vessel stenosis < 50% counted as zero. The results showed that the MACE rate at 1‐year follow‐up was higher in the CFR < 1.6 group with or without revascularization therapy, while the group with low CADPI and CFR≥1.6 had the highest rate without cardiovascular events. Multiple regression analysis showed that CFR was a more important predictor of the risk of MI and heart failure than CADPI with a hazard ratio (HR) of 2.02 and 1.17, respectively.^11^ The Women's Ischemia Syndrome Evaluation (WISE) study showed that patients with INOCA had a poor prognosis, and a decreased CFR was a strong independent predictor of MACE.^12^ The 2015−2018 international multicenter enrollment cohort study launched by the US COVADIS project team showed that in 686 patients (women 64%) with MVA pectoris excluding epicardial vessel stenosis > 50%, coronary microvascular spasm was the most common (42%), followed by decreased CFR (35%), increased microvascular resistance (14%), and slow coronary flow (6%). The incidence of composite endpoints, including cardiovascular death, nonfatal MI, nonfatal stroke, and hospitalization for heart failure or angina, was 7.7% after 1 year of follow‐up.^13^

In a retrospective study, the results of CAG performed in 1600 patients in 6 centers of China were analyzed, and the prevalence rate of INOCA was about 20%, and female was identified as a risk factor of INOCA.^14^ In a systematic review and meta‐analysis that included 56 studies and 14,427 patients with INOCA, microvascular function was evaluated using invasive or noninvasive diagnostic methods, and the results showed that 41% of patients had coronary microvascular dysfunction, 40% had epicardial coronary spasm, and 24% had microvascular spasm. In addition, the incidence of coronary artery microvascular dysfunction in women was 1.45 times higher than that in men.^15^

#### 2.3.2 CMVD associated with MI

CMVD‐associated MI can be divided into two subtypes: CMVD associated with MIOCA and CMVD associated with MINOCA. The incidence of adverse cardiovascular events significantly increases when MIOCA coexists with CMVD. MINOCA is more common in women, and CMVD is an important pathogenic factor for MINOCA.

A systematic review and meta‐analysis analyzed cardiovascular endpoints in 1094 patients (women 18.2%) with ST‐segment elevation MI (STEMI) in six studies conducted between 2013 and 2020. The endpoints were all‐cause death, nonfatal MI, and combined cardiovascular events with hospitalization for heart failure, and follow‐up ranged from 6 months to 7 years. Using an index of microcirculatory resistance (IMR) > 40 or hyperemic microvascular resistance (HMR) > 3 mmHg/cm/s as an indicator for severe CMVD, the results showed that severe CMVD had a worse prognosis, with an HR of 3.42 compared with nonsevere CMVD.^16^ A meta‐analysis of 46 studies showed an incidence of MINOCA of 6% (patients’ median age 55 years), which was more common in young women without hyperlipidemia, and 12‐month all‐cause mortality was lower in patients with MINOCA than MIOCA (4.7% vs. 6.7%).^17^ In the absence of culprit plaque rupture or erosion, microvascular dysfunction may have a critical role in MINOCA. Forty‐four female patients with MINOCA underwent cardiovascular magnetic resonance (CMR), and late gadolinium enhancement imaging was observed in 59% of these patients, indicating myocardial ischemia caused by microvascular dysfunction.^18^ In addition, 96 patients with MINOCA underwent acetylcholine stress test, and one‐third patients were found to have microvascular spasm.^19^

#### 2.3.3 CMVD associated with coronary revascularization

CMVD associated with coronary revascularization is mainly manifested with no‐reflow, which is more common and the prognosis is worse in women than in men. Long‐term MACE increased significantly in patients with CMVD after elective percutaneous coronary intervention (PCI). Evidence of coronary microvascular dysfunction was also found in both short‐term and long‐term follow‐up of patients undergoing coronary artery bypass grafting (CABG).

A 2010–2016 Italian‐registration study included 2596 patients with STEMI (women 25.9%) who underwent primary PCI (PPCI). The primary endpoint was 30‐day mortality. The results showed that women had a higher rate of primary endpoint than men (4.8% vs. 2.5%, odd ratio = 2.0). Multiple regression analysis showed that women were more likely to develop no‐reflow (postprocedural thrombolysis in MI (TIMI) flow grade 0–2, HR = 1.68).^20^ An international, multicenter observational study recruited 572 patients with stable CAD who underwent coronary interventional therapy, and IMR ≥ 25 was defined as coronary microvascular dysfunction. After 4 years of follow‐up, the cumulative MACE, including perioperative MI, recurrent MI, all‐cause mortality and revascularization, was significantly higher in patients with a high IMR than in those with a low IMR (HR = 1.56, *p* = 0.001).^21^ In a retrospective study, 341 patients who had previously received CABG were followed for 638 days. After adjusting for known prognostic factors (regional ischemia, infarction), both stress myocardial blood flow (MBF) and myocardial perfusion reserve (MPR) independently predicted the composites of all‐cause mortality and MACE. The adjusted HR for 1 mL/g/min of decrease in stress MBF was 2.56 (95% CI: 1.45–4.35) and for 1 unit of decrease in MPR was 1.61 (95% CI: 1.08–2.38).^22^ Spyrou et al.^23^ measured MBF at rest and during dipyridamole‐induced hyperemia using PET in eight patients who underwent CABG, and found that coronary microvascular dysfunction was present at 1 and 6 months postoperatively, and the recovery was slow.

#### 2.3.4 CMVD associated with non‐atherosclerotic heart disease

CMVD has been reported in a variety of patients with non‐atherosclerotic heart disease. CMVD with cardiac hypertrophy is common in hypertrophic cardiomyopathy (HCM),^24^ aortic stenosis (AS),^25^ cardiac amyloidosis,^26^ heart failure with preserved ejection fraction (HFpEF),^27^ and Anderson–Fabry disease. On the other hand, CMVD without myocardial hypertrophy is common in stress cardiomyopathy,^28^ diabetic cardiomyopathy,^29^ dilated cardiomyopathy,^30^ and autoimmune diseases.^31^, ^32^ However, most of these associations came from small sample studies.

## 3 PATHOGENETIC MECHANISM OF CMVD

### 3.1| Structural and physiological characteristics of the coronary artery

Coronary artery is a continuous vascular network consisting of four segments^33^: (1) epicardial coronary artery: the luminal diameter of the epicardial coronary arteries is 0.5–5 mm, which act as a capacitance vessel and offer little resistance to coronary blood flow (CBF); (2) prearteriole: the luminal diameter of the prearterioles is 100–500 μm, which are mainly responsible for regulating coronary artery perfusion pressure and exert a major effect on coronary blood flow resistance. The prearterioles can be further divided into two segments: the proximal prearterioles (150–500 μm) are more responsive to changes in flow, and the distal prearterioles (100–150 μm) are more sensitive to pressure variations; (3) arteriole: the luminal diameter of arterioles is <100 μm, which are sensitive to the change of myocardial metabolite concentration and play a major role in matching myocardial blood supply with oxygen consumption^34^; (4) capillary: myocardial capillaries are composed of monolayer endothelial cells, which provide 90% of the blood supply to the myocardium, and are mainly responsible for the exchange of myocardial oxygen, nutrients, and metabolites.^35^ Prearterioles, arterioles, and capillaries constitute coronary microcirculation. Pressure gradually decreases as blood flows from the aorta to the coronary capillaries: 10% pressure reduction occurs in epicardial coronary arteries, 30% in prearterioles, 40% in arterioles, and 20% in capillaries and venules. Therefore, the coronary microcirculation plays a crucial role in regulating coronary perfusion pressure and blood flow.^36^

### 3.1 Regulatory mechanism of CBF

#### 3.1.1 Regulatory mechanism of coronary vasomotor

Vasomotor function in different coronary segments is regulated by different mechanism:

(1) *Flow‐mediated vasodilation*: This mainly occurs at the epicardial coronary artery and prearteriole levels. When the shear stress of blood flow increases, endothelial cells release nitric oxide (NO), endothelium‐dependent hyperpolarization factors (EDHFs) and prostacyclin to induce vasodilation.

(2) *Coronary blood flow autoregulation*: Under the condition of unchanged myocardial metabolism, the CBF remains consistent despite a wide variation of coronary perfusion pressure (60–100 mmHg). This phenomenon is called automatic regulation, and the mechanism may involve myogenic reaction at the distal prearterioles, that is, when the coronary perfusion pressure rises, the prearterioles contract and vice versa.

(3) *Myocardial oxygen consumption*: The maximum oxygen supply of coronary artery can be five times larger than that in the resting state due to a fully dilation of coronary resistance vessels to meet an increased demand of myocardial oxygen consumption, which has been termed “functional hyperemia.” A variety of mediators are involved in this mechanism, including neurotransmitters in circulation, NO, EDHFs, prostacyclin, and endothelin produced by vascular endothelial cells, histamine, kinin, and interleukin produced by vascular adventitial cells, and thromboxane A2 and serotonin produced by platelets.

(4) *Myocardial metabolites*: Hypoxia can induce adenosine production and stimulate the adenosine A2 receptor of smooth muscle cells to dilate the coronary artery. During myocardial ischemia or hypoxia, the accumulation of myocardial metabolites first dilates the coronary arterioles resulting in decreased coronary artery resistance and perfusion pressure in the prearterioles, which triggers a myogenic response of the coronary prearterioles, further dilates blood vessels, and increases the shear stress of coronary blood flow. The increased shear stress may trigger blood flow‐mediated vascular dilation of the epicardial coronary arteries and larger prearterioles. This cascade regulatory mechanisms ensure that other mechanisms can compensate when one regulatory mechanism fails in pathological conditions, thus avoiding myocardial ischemia.^37^

#### 3.1.2 CFR

CFR is defined as the ratio of CBF or MBF during maximum coronary dilation to the corresponding indicators in the resting state. It is an overall indicator of the reserve function of the entire coronary system. CFR is affected by four factors: resting CBF, the cross‐sectional area of resistance vessels per unit volume of the myocardium, extravascular pressure, and coronary perfusion pressure. In clinical practice, factors affecting coronary or MBF at rest are as follows: age, sex, myocardial oxygen consumption (heart rate and blood pressure), medications, abnormal vascular endothelial function, and myocardial fibrosis. The following factors are known to affect coronary or MBF in the hyperemia state: age, inadequate coronary dilation, coronary perfusion pressure, caffeine, and its derivatives which may counteract the effects of adenosine or dipyridamole, microvascular anatomical remodeling, increased microvascular tension, increased extravascular tension, abnormal vascular endothelial function, and myocardial fibrosis.^38^

### 3.2 Structural abnormalities of the coronary microcirculation

Microvascular remodeling and stenosis are consistently documented in patients with HCM and hypertensive heart disease.^39^ The pathogenesis may involve genetic factors such as gene polymorphisms of the renin–angiotensin–aldosterone system (RAAS), peroxisome proliferator‐activated receptors and endothelin, hemodynamic changes such as a high shear stress induced by blood pressure elevation, and neurohumoral mechanisms including RAAS activation in hypertension, high expression of cytokines, adhesion molecules, and endothelin, as well as inflammatory cell accumulation. All these mechanisms may result in endothelial dysfunction and proliferation of fibroblast and vascular smooth muscle cells, leading to varying degrees of intimal and medial thickening, perivascular fibrosis, intramural arteriolar remodeling and stenosis, and capillary rarefaction. Ultimately, these structural abnormalities cause increased coronary microvascular resistance and decreased CBF.^40^, ^41^, ^42^

### 3.3 Microvascular obstruction

Coronary microvascular obstruction (MVO) is commonly seen in emergency PCI and intervention of stenotic saphenous vein graft.^43^ The following mechanisms are involved: (1) acute coronary artery occlusion causes acute hypoxia of endothelial cells and reduced flow shear stress, leading to a series of biochemical and metabolic changes, such as increased anaerobic glycolysis, intracellular acidosis and calcium overload, and release of reactive oxygen species and inflammatory cytokines. These abrupt changes may lead to endothelial swelling and disruption, increase in vascular permeability, loss of vasodilator response, and constriction of smooth muscle cells, and finally cause injury and obstruction of the coronary microcirculation. In addition, myocardial reperfusion injury may further aggravate microvascular damage; (2) obstruction of distal coronary microvessels due to microthrombi and debris released from atherosclerotic plaques during various intervention procedures^44^; (3) microvascular compression due to myocardial cell apoptosis, myocardial edema and inflammation, which may lead to erythrocyte extravasation and intracardiac hemorrhage^45^; (4) coronary microvascular constriction caused by microthrombi or aggregated leukocyte‐platelet.^46^

### 3.4 Functional abnormalities of the coronary microcirculation

#### 3.4.1 Impaired endothelium‐dependent vasodilation

Endothelium‐dependent vasodilation dysfunction is more common in patients with risk factors for cardiovascular disease (such as diabetes, dyslipidemia, obesity, and smoking) or atherosclerosis. Prostaglandins, NO and EDHF synthesized and released by endothelial cells play a key role in regulating vascular tension, and endothelium‐dependent coronary microcirculation dysfunction may be attributed to the decreased production or attenuated role of these vasodilators.^47^ EDHFs are more important than NO in the pathogenesis of CMVD due to their function of dilating coronary resistance vessels.^47^, ^48^

#### 3.4.2 Impaired endothelium‐independent vasodilation

In patients with diabetes, metabolic syndrome, dyslipidemia, hypertension, obesity, smoking, renal disease, and cardiomyopathy, the coronary artery dilation response to papaverine, adenosine, or dipyridamole is weakened, suggesting impaired endothelium‐independent vasodilation.^49^ Consequently, these abnormalities may lead to decreased reactivity of coronary arteries to vasodilating substances, and declined CFR.

#### 3.4.3 Microvascular spasm

Microvascular spasm is commonly seen in patients with angina or MINOCA.^19^, ^50^ Coronary microvascular spasm is primarily attributed to myosin light chain phosphorylation induced by Rho kinase, increased secretion of vasoconstrictive agonists (such as endothelin and serotonin), and increased vasoconstrictive reactivity of coronary microvessels due to inflammatory states.^51^, ^52^, ^53^

#### 3.4.4 Dysfunction of cardiac sympathetic neurons

In the resting state, sympathetic nerves have a limited capacity to regulate coronary vasomotor function. During exercise, however, sympathetic nerves may release norepinephrine and regulate coronary tone. β‐adrenergic stimulation activates β2‐receptors in the coronary arteries and promotes coronary artery dilation to compensate for increased myocardial oxygen consumption. When endothelial dysfunction occurs due to coronary atherosclerosis, α1‐adrenergic‐mediated vasoconstriction is more prominent, resulting in decreased CBF and myocardial ischemia.^54^

**FIGURE 1:**
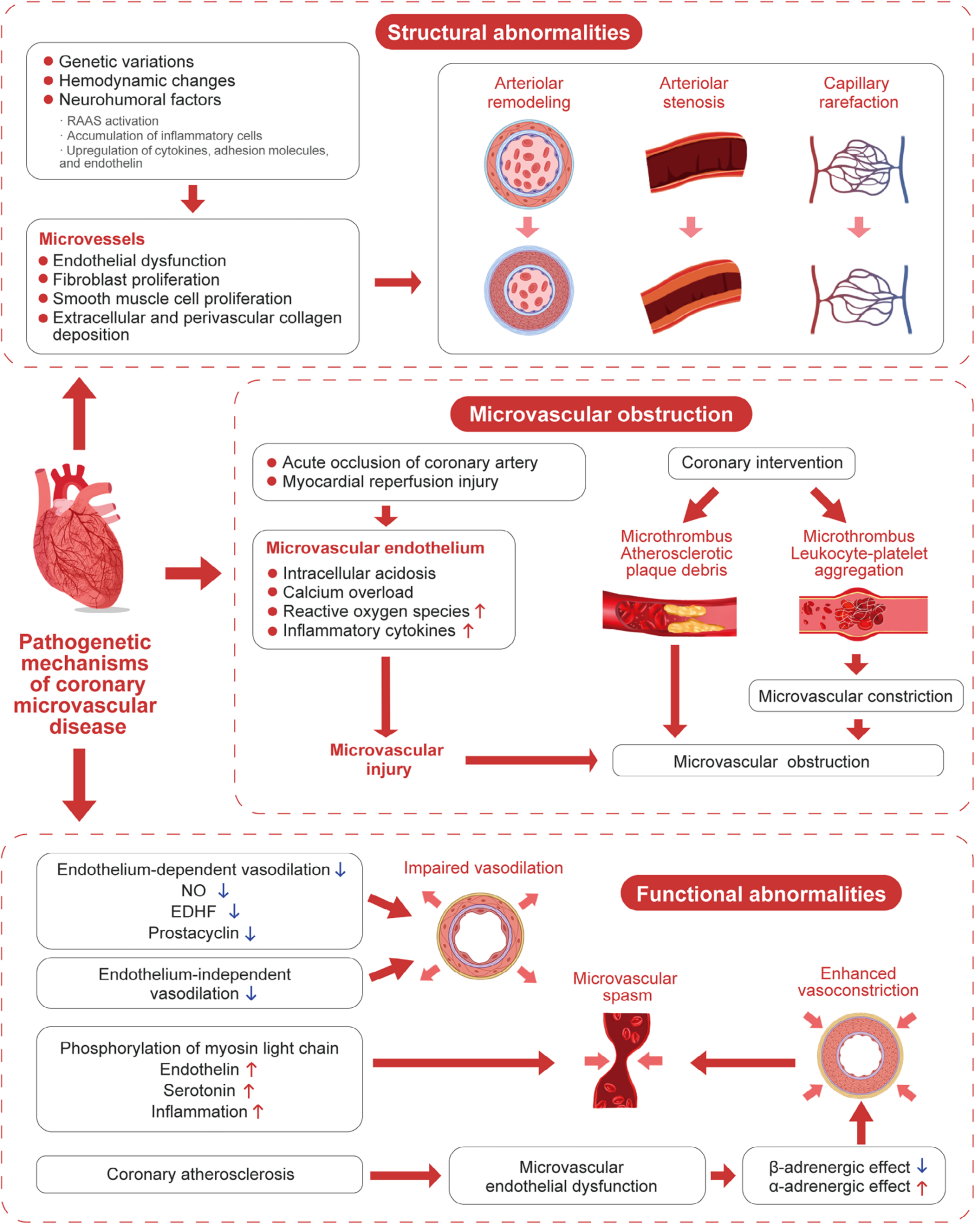
Pathogenetic mechanisms of coronary microvascular disease. Three key mechanisms are involved in the pathogenesis of CMVD. The first one is structural abnormalities of the coronary microcirculation. Genetic variations, hemodynamic changes, and neurohumoral factors may result in endothelial dysfunction, proliferation of fibroblasts and vascular smooth muscle cells and collagen deposition, leading to microvessel remodeling and stenosis, and capillary rarefaction. The second one is microvascular obstruction. Acute coronary artery occlusion and myocardial reperfusion injury elicit a series of biochemical and metabolic reactions, resulting in microvascular injury and obstruction. Coronary interventions may produce microthrombus and plaque debris, leading to microvascular occlusion. The third one is functional abnormalities of the coronary microcirculation. Impaired endothelium‐dependent or endothelium‐independent vasodilation, microvascular spasm, and dysfunction of cardiac sympathetic neurons may lead to enhanced vasoconstriction of microvessels. The figure was original and created using photoshop software. EDHF, endothelium‐dependent hyperpolarization factors; NO, nitric oxide.

It should be noted that microvascular dysfunction may be a systemic disease, and patients with CMVD may also have cerebral, retinal or renal small vessel disease.^55^ The pathogenetic mechanisms of CMVD are summarized in Figure 1.

## 4 DIAGNOSTIC TECHNIQUES FOR CMVD

### 4.1 Vasoactive drugs for evaluating coronary microvascular function

Coronary microvascular function is frequently assessed by evaluating the coronary microvascular response to vasodilators, because of the limitation of current imaging techniques in displaying morphological changes of coronary microvasculature. CFR is a commonly used index. Vasodilators include endothelium‐independent vasodilators, which primarily act on vascular smooth muscle cells, and endothelium‐dependent vasodilators, which primarily act on vascular endothelial cells.

(1) *Adenosine*: Adenosine is the most commonly used endothelium‐independent vasodilator for assessing coronary microvascular function. Adenosine receptors are G protein‐coupled glycoproteins classified as four types: A1, A2A, A2B, and A3. The effect of coronary dilation is related to A2 receptors, while the effect on ischemia–reperfusion injury is related to A2A receptors. Adenosine can be administered intravenously at a dose of 140 μg/(kg·min) or intracoronarily at a dose of 2–16 μg/(kg·min) with an injection duration of 1.5–6.0 min. The advantage of adenosine is a very short half‐life of only 10 s. The common adverse effects include atrioventricular block, sinoatrial block, and bronchospasm. Once occurring, however, these side effects usually disappear quickly.^56^

(2) *Dipyridamole*: Dipyridamole acts by inhibiting adenosine degradation, with a pharmacological effect similar to adenosine. Dipyridamole is diluted with glucose into 5 or 10% solution and administrated intravenously at a dose of 0.56–0.84 mg/kg. After administration of adenosine or dipyridamole, a CFR value of <2.5 indicates abnormal coronary microvascular dilative function.^57^ In clinical practice, CFR < 2.0 is commonly regarded as a cutoff value for the diagnosis of microvascular dysfunction.^58^

(3) *Acetylcholine*: Acetylcholine is a commonly used vasodilator for evaluation of endothelium‐dependent coronary microvascular function. It works by two different mechanisms: (a) stimulating endothelial cells to release NO, which results in vasodilation, and (b) binding to muscarinic acetylcholine receptors, which activates smooth muscle cells and results in vasoconstriction. When vascular endothelial function is normal, the vasodilative effect of acetylcholine predominates. However, in the setting of endothelial dysfunction, the vasoconstrictive effect becomes prominent, resulting in vasospasm. Acetylcholine diluted in solution is injected into coronary artery at an incremental dose.^59^ One minute after injection of acetylcholine, CAG is performed immediately, and the diameter of the coronary artery is measured by quantitative CAG to determine whether epicardial coronary spasm is present. Anginal symptoms and changes of electrocardiogram (ECG) are monitored simultaneously. If angina pectoris occurs or ischemic ST‐T changes appear without detectable epicardial coronary artery spasm after injection, CMVD can be diagnosed. At the same time, nitroglycerin or nicorandil should be immediately injected into coronary artery to relieve coronary microvascular spasm.

(4) *Regadenoson*: Regadenoson is a selective agonist of adenosine A2A receptor. It dilates coronary arteries and increases coronary blood flow by selectively activating adenosine A2A receptors. The advantages of regadenoson lie in its selective dilatation of coronary arteries, rapid onset of pharmacological effect and a fixed dose recommended without the need for adjustment for patient's body weight. Regadenoson is administered at a bolus injection of 0.4 mg/5 mL in 10 s. Currently, regadenoson has been widely used as the drug of choice to evaluate coronary microcirculation function^5^ in a variety of pharmacological stress tests, including ECG, echocardiography, PET, CMR, and computed tomography (CT).^60^

(5) *Nicorandil*: Nicorandil is a nitrate and adenosine triphosphate (ATP)‐sensitive potassium channel opener, which activates guanylate cyclase in cells and relax vascular smooth muscles, and dilates both epicardial coronary arteries and coronary microvessels without affecting myocardial contractility, blood pressure, myocardial oxygen consumption and heart rate. Its pharmacological effects and duration of maximum hyperemia are dose dependent. Nicorandil is injected into coronary artery at a bolus injection dose of 2–4 mg.^61^, ^62^ For fractional flow reserve (FFR) and IMR measurement, intracoronary injection of nicorandil at a dose of 3 mg is a suitable alternative to ATP. The advantages of nicorandil include direct intracoronary injection, maximum hyperemia achieved with short time, sufficient duration of hyperemia, few side effects which subside quickly after drug withdrawal. However, most studies examining microcirculation function before and after nicorandil injection were conducted in a single center, and the sample sizes were limited.

### 4.2 Noninvasive techniques for evaluating coronary microvascular function

#### 4.2.1 Transthoracic Doppler echocardiography

Coronary blood flow of the distal left anterior descending artery (LAD) can be clearly visualized by transthoracic Doppler echocardiography (TTDE) in more than 90% of patients, and after intravenous injection of ultrasound contrast, the success rate of LAD flow imaging can be increased to 100%.^63^ The success rate for imaging the posterior descending artery ranges from 54% to 86%, and for imaging the left circumflex flow is lower. TTDE allows measurement of the maximal diastolic flow velocity in LAD at rest and during stress test with adenosine, dipyridamole, or regadenoson, and a ratio of stress to rest velocity can be calculated, which is called coronary flow velocity reserve (CFVR).^64^ In the absence of flow‐limited stenosis of LAD, CFVR is a reliable measure of coronary microvascular function, and the cutoff of ≤2–2.5 indicates impaired coronary microvascular function.^4^

TTDE is a noninvasive, time‐saving, feasible, repeatable, relatively inexpensive, and nonradiative technique. However, it requires extensive technical training and can only be used when the LAD flow is clearly visualized.^65^

#### 4.2.2 Myocardial contrast echocardiography

Myocardial contrast echocardiography (MCE) is a noninvasive technique for evaluating coronary microvascular function based on myocardial backscatter signals detected by ultrasound technology after intravenous injection of microbubble contrast agent.^66^ CFR at the myocardial level can be derived by calculating the ratio of MBF during exercise or pharmacological stress to that at rest. MBF acquired by MCE correlated well with MBF by PET in clinical patients,^67^ and CFR measured by MCE during stress test has been used to assess the stenotic severity of epicardial coronary arteries and detect MVO after MI.^68^ However, there are few clinical studies of this technique to evaluate CMVD.

MCE is a noninvasive, feasible, and relatively inexpensive technique that can be performed at bedside and does not expose patients to radiation. However, image quality of MCE is operator dependent and affected by obesity, respiratory movement, and pulmonary disease. In addition, uneven distribution of ultrasound intensity in a two‐dimensional sector may lead to attenuated myocardial contrast signals in the bilateral edges and far field, with a false positive finding of myocardial hypoperfusion.

#### 4.2.3 Single‐photon emission CT

This technique uses a tracer labeled with thallium‐201 (^201^Ti) or technetium‐99m (^99m^Tc) to record myocardial radioactivity at rest and during stress test. Signs of segmental hypoperfusion, perfusion defect, or perfusion redistribution can be detected, which are indicative of CMVD in the absence of significant epicardial coronary artery stenosis. The newly developed three‐dimensional single‐photon emission CT (SPECT) technology can be used for quantitative assessment of MBF, but the accuracy is affected by respiration and heartbeat.^69^ SPECT/CT using a low‐dose CT scan can overcome SPECT imaging attenuation, optimize the anatomical co‐localization of the heart, and perform partial correction of volume effect. Thus, dynamic SPECT imaging technology combined with CT has become a new method for measuring MBF. A heart‐specific SPECT using new semiconductor and cadmium zinc telluride crystal as a tracer has significantly improved spatial resolution and sensitivity of cardiac imaging, shortened scanning time, and reduced radiation dose.^70^

SPECT has a high diagnostic sensitivity and negative predictive value in evaluating coronary microvascular function, but it cannot measure CFR quantitatively using conventional techniques and is associated with radiation exposure and a low spatial resolution.

#### 4.2.4 PET

This technique uses an intravenously injected radionuclide‐labeled tracer, such as ^15^O, ^13^N, and ^82^Rb, to continuously monitor the radiation activity in the blood and myocardium. By recording the time‐radioactivity curve reflecting the dynamic changes in radionuclide uptake of the left ventricular cavity and myocardium, MBF per gram of myocardium per minute can be calculated. When the myocardial load increases, the myocardial oxygen consumption increases, and the MBF will increase by three to four times. However, in the setting of coronary microcirculation dysfunction, MBF cannot meet the myocardial oxygen demand, leading to myocardial ischemia. The ratio of MBF measured after coronary vasodilator administration to MBF at rest is equivalent to CFR. Currently, MBF and CFR measured by PET are the gold standard for the noninvasive diagnosis of myocardial ischemia, and PET has a high reproducibility within a certain coronary blood flow range (0.5–6 mL/g/min).^71^, ^72^

Recent development of PET/CT technology has partially overcome the attenuation effect of PET through anatomical correction by CT.^73^ In addition, PET/magnetic resonance imaging (MRI) technology is expected to reduce PET attenuation through MRI correction, thereby improving its accuracy.^74^ Furthermore, the development of 3D‐PET technology is expected to reduce radiation dose and increase the accuracy of PET scanning.^75^ Compared with SPECT, PET provides higher spatial resolution and better attenuation correction, resulting in enhanced image quality. In comparison with technetium‐99m‐labeled SPECT perfusion tracers, commonly used PET tracers have lower radiation exposure and better mobility in the myocardial microcirculation.^76^

The advantages of PET are an accurate quantification of MBF and CFR for both resting and hyperemic conditions, accurate evaluation of myocardial perfusion, and availability of multiple tracers. Its disadvantages are time‐consuming, a high cost, a limited spatial resolution and radionuclide exposure.

#### 4.2.5 CMR

CMR is a noninvasive imaging technique that can simultaneously assess cardiac anatomy, morphology, function and myocardial perfusion.^68^ The characteristic of CMR is to perform first‐pass perfusion imaging of the myocardium based on the T1 relaxation properties of gadolinium as a contrast agent. In T1‐weighted images, normally perfused myocardium shows a uniform increase in the first‐pass signal intensity of the gadolinium. In the presence of microcirculation dysfunction, however, the signal intensity in the ischemic zone increases slowly relative to that at the adjacent normal myocardial segments, yielding a visible low signal area. MBF (mL/min/g) at the resting and hyperemic states can be measured from the intensity curves in myocardial area of interest.

A semi‐quantitative MPR index (MPRI) can be obtained from routine CMR imaging, but a decreased MPRI may be caused by an increased resting myocardial perfusion or impaired microvascular function. The CMR myocardial perfusion measurement sequence corrected for respiratory motion, which is being tested in clinical trials, allows patients to breathe freely and quantifies MBF pixels during postprocessing.^77^ Fully quantitative MBF measured by CMR has been shown to correlate well with MBF determined by PET.^78^ Myocardial perfusion imaging by CMR during adenosine stress test is of diagnostic value, which has been used in clinical studies to assess coronary artery stenosis, detect CMVD and risk‐stratify clinical patients.^79^, ^80^

There are a number of advantages of CMR including a high feasibility, high spatial resolution, absence of radiation exposure and signal attenuation, simultaneous detection of myocardial function, structural morphology, myocardial edema and myocardial perfusion, accurate differentiation of myocardial ischemia caused by coronary arterial stenosis or microvascular disfunction, as well as accurate evaluation of myocardial perfusion, coronary artery resistance and diastolic filling time. With these advantages, CMR has gradually become the gold standard for noninvasive evaluation of myocardial ischemia. The disadvantages of CMR are common subendocardial artifacts that can affect visual image analysis and MBF calculations,^77^ and adverse reactions caused by conventional gadolinium contrast in patients with renal insufficiency.

#### 4.2.6 CT perfusion imaging

CT perfusion (CTP), which is based on computed tomographic angiography (CTA), has emerged as a novel noninvasive and “one‐stop” solution for the comprehensive assessment of both anatomy and physiology of epicardial coronary arteries.^81^ The CTP protocol includes resting scanning (coronary CTA), and stress scanning (pharmacological stress). The former is used to reliably rule out any significant epicardial coronary stenosis, and the latter is used to assess microvascular function by qualitative or quantitative evaluation of MBF distribution based on the difference in CT values when blood flow goes through different myocardial segments. Stress scanning of CTP can be further divided into two modes^82^: first, static CTP, which acquires images of only one cardiac cycle when the contrast first passes through myocardium, and a visual qualitative assessment of myocardial perfusion is performed according to CT values in different myocardial segments; and second, dynamic CTP, which continuously acquires images when the contrast goes through myocardium to create a time‐CT value curve and thereby calculate MBF and myocardial blood volume to achieve quantification of myocardial perfusion.^83^

Currently, CTP is the only noninvasive modality that allows simultaneous assessment of epicardial coronary arteries and coronary microcirculation. The accuracy of CTP to identify microcirculatory perfusion defects is comparable to that of SPECT.^84^ In addition, CTP has a low cost and is easy to accept by most patients. In patients in whom precise evaluation of coronary artery morphology is precluded due to coronary stenting, severe coronary calcification and imaging artifacts, CTP enables functional assessment of myocardial perfusion, thus breaking the diagnostic limitations of conventional coronary CTA.^85^ Furthermore, CTP offers several advantages over CT‐FFR, which focuses only on a focal lesion. The disadvantages of CTP are an increased contrast dose and radiation exposure. The lack of a recognized cutoff value also limits its clinical use. The advantages, disadvantages, levels of evidence, and grades of recommendation of noninvasive techniques for assessing microvascular function are listed in Table 5.

**TABLE 5:** Noninvasive techniques for evaluating coronary microvascular function.

Technique	Method	Agent	Parameter	Diagnostic threshold	Advantages	Limitations	Clinical significance	Class of recommendation	Level of evidence	References
TTDE	Pulsed‐wave Doppler on the proximal LAD artery	Vasodilator: • Adenosine • Dipyridamole • Regadenoson	CFVR	CFVR < 2	• Bedside • Safe • Readily available • Inexpensive • Radiation‐free	• Limited to the LAD region • Operator dependent • Technical pitfalls (poor acoustic window in obesity and lung diseases) • Obstructive CAD needs to be excluded • Very limited data with use in nonobstructive CAD	Only applicable in patients without significant LAD obstructive stenosis	IIa	C	^64^
MCE	Backscatter signal of microbubbles from the microvasculature is detected using low‐power harmonic ultrasound	Vasodilator • Adenosine • Dipyridamole • Regadenoson Contrast agents: • Sulfur hexafluoride microbubbles for injection	CFR	CFR < 2	• Readily available • Bedside • Radiation‐free • Safe • Good correlation with MBF by PET	• Operator dependent • Technical pitfalls (poor acoustic window in obesity and lung diseases) • Obstructive CAD needs to be excluded • Very limited data with use in nonobstructive CAD • Unavailability of validated commercial software for CFR quantification	Allows evaluation of CFR in different myocardial segments or regions	I	A	^112^, ^113^
SPECT	Distribution of radionuclides indicates myocardial perfusion for both resting and stress conditions	Vasodilator: • Adenosine • Dipyridamole • Regadenoson Tracers: • ^201^Ti • ^99m^Tc	MBF, CFR	CFR < 2	• High sensitivity and specificity for detection of ischemia • Less expensive than PET	• Limited ability for absolute quantification of MBF • Operator dependent • Limited spatial resolution • Radiation exposure	• Distinguishes between CMVD and epicardial coronary lesions, based on tissue characterization • Allows evaluation of LV function	IIa	B	^114^
PET	Radioactivity curves of the distribution of radionuclides are dynamically recorded	Vasodilator: • Adenosine • Dipyridamole • Regadenoson Tracers: • ^13^N • ^82^Rb • ^15^O	MBF, CFR	CFR < 2	• Gold standard for noninvasive and quantitative assessment of coronary microvascular function • Reproducibility	• High costs • Operator dependent • Limited availability • Limited spatial resolution • Radiation exposure • Limited diagnostic ability for microcirculation dysfunction with obstructive CAD	• Comprehensive and accurate evaluation of MBF and CFR for both resting and stress conditions • Multiple available tracers	I	A	^115^, ^116^, ^117^
CMR	Myocardial signal intensity is recorded during the first‐pass contrast uptake for both resting and stress conditions	Vasodilator: • Adenosine • Dipyridamole • Regadenoson Contrast agent: • Gadolinium‐based	MPR, MPRI, MBF, CFR	MPRI < 2, MBF < 2.25 mL/g/min, CFR < 1.5–2	• Radiation‐free • Excellent spatial resolution • Coronary territories can be evaluated simultaneously • Tissue characterization	• High costs • Time‐consuming • Poor patient compliance • Limited availability • Limited ability for absolute quantification of MBF • Contraindicated in patients with severe renal disease, claustrophobia, arrhythmias, and implanted devices	• Applicable in patients with unobstructed coronary arteries and suspected primary CMVD • Distinguishes between CMVD and epicardial coronary lesions, based on tissue characterization	I	A	^79^, ^118^, ^119^
CTP	Signal intensity of contrast medium is recorded at rest and stress conditions using dynamic first‐pass effect	Vasodilator: • Adenosine • Dipyridamole • Regadenoson Contrast agent: • Iodine‐based	MBF, MPR	MPR < 2	• Combination of coronary anatomy and myocardial perfusion as “one‐stop” solution • Simultaneous evaluation of all coronary territories • CCTA‐derived FFR	• Radiation exposure • Risk of kidney disease • Lacking standard cutoff for MBF • Still under investigation	Comprehensive work‐up of suspected CAD and/or CMVD	IIb	C	^83^, ^84^

Abbreviations: CAD, coronary artery disease; CCTA, coronary computed tomography angiography; CFR, coronary flow reserve; CFVR, coronary flow velocity reserve; CMR, cardiovascular magnetic resonance; CMVD, coronary microvascular disease; CTP, computed tomographic perfusion; FFR, fractional flow reserve; LAD, left anterior descending branch; LV, left ventricle; MBF, myocardial blood flow; MCE, myocardial contrast echocardiography; MPR, myocardial perfusion reserve; MPRI, myocardial perfusion reserve index; PET, positron emission tomography; SPECT, single‐photon emission computed tomography; TTDE, transthoracic Doppler echocardiography.

### 4.3 Invasive techniques for evaluating coronary microvascular function

#### 4.3.1 Coronary angiography

Two methods are available to evaluate the epicardial coronary artery flow by CAG: (1) TIMI flow grading: TIMI (grades 0–3) is widely used to evaluate the patency of epicardial coronary artery, but is only a semi‐quantitative parameter and cannot reflect coronary microvascular function^86^; (2) TIMI flow frame count (TFC): TFC is the number of frames from the beginning of coronary artery imaging to the standardized distal marker imaging, which overcomes the shortcomings of semi‐quantitative nature of TIMI flow grading. However, it does not directly reflect coronary microvascular flow.^87^

“Slow coronary flow” is an angiographic phenomenon characterized by a delayed visualization of distal vessels of a nonobstructive coronary artery, which is considered a manifestation of CMVD. The definition for “slow coronary flow” varies between different studies, some using TIMI flow grades 1−2 while others using a modified TIMI flow count of >25 frames.^4^

Several indexes measured by CAG have been proposed to assess coronary microvascular function based on the speed of myocardial opacity after CAG, such as TIMI myocardial blush grade (TMBG), myocardial blush grade (MBG), and TIMI myocardial perfusion frame count (TMPFC). TMBG is classified into grades 0–3 according to the duration of ground glass appearance of the myocardium after CAG, which can be used as a semi‐quantitative measure to reflect the patency of coronary microvessels.^88^ MBG is classified into grades 0–3 based on changes in myocardial contrast density after the contrast enters the myocardial tissue, which can be used as a semi‐quantitative index to reflect the perfusion status of the coronary microcirculation.^89^ TMPFC is an index put forward by Chinese researchers to quantitatively evaluate myocardial reperfusion immediately after PCI by measuring the number of frames from the onset of myocardial blushing to contrast emptying from the myocardium.^90^ Studies have shown that an increased TMPFC predicted microvascular dysfunction defined by MRI gadolinium imaging after PCI.^91^

An advantage of selective coronary angiographic techniques is an immediate evaluation of coronary microvascular function after PCI, with a high technical feasibility and analytical simplicity. However, these indices are affected by coronary perfusion pressure and heart rate, and cannot reflect CFR.

In a proportion of patients with myocardial ischemia, coronary motor disorder at epicardial and/or microvascular levels may play a role, but a definitive diagnosis requires the use of coronary acetylcholine provocative test during CAG.^92^, ^93^ Accumulating clinical experience with this provocative test has proven its safety and usefulness in identifying patients at a high risk of future clinical events.^94^

#### 4.3.2 Thermodilution technique

(1) *Bolus thermodilution*: The conventional thermodilution technique is the bolus thermodilution method. It is based on the principle of the Fick method, where cold saline at a given temperature and injection rate is bolus injected as a tracer through a catheter into the ostium of a coronary artery, and the blood temperature is measured at the distal coronary artery. The magnitude of blood temperature drop indicates the degree of tracer dilution, which is proportional to CBF, and thus CBF can be estimated. The time required for cold saline to leave the guide catheter to reach the guidewire sensor in distal coronary artery can be recorded from the temperature dilution curve, that is, the mean transient time (*T*). The *T* value is inversely proportional to CBF, and the ratio of *T* values recorded at baseline and maximal hyperemia is CFR (*T* baseline/*T* hyperemia).^95^

Index of microvascular resistance (IMR) has been recognized as a feasible index to evaluate the microvascular function distal to a stenotic lesion, which is defined as a ratio of distal coronary pressure to distal coronary flow, that is, the product of the distal coronary pressure (Pd) and mean transit time (*T*) of a saline bolus during maximal hyperemia. Pd and T are measured by a pressure guidewire equipped with a temperature sensor.^96^ IMR is independent of epicardial vascular function and able to specifically evaluate microvascular function with a good reproducibility.^96^, ^97^, ^98^

Of the bolus‐thermodilution method, large intraobserver variability and overestimated CFR at higher values were observed due to manual rapid injection of saline. It is also susceptible to subjectivity with an alternative *T* value. Additionally, intravenous or intracoronary adenosine is associated with multiple side effects.

(2) *Continuous thermodilution*: Recently, a novel method based on continuous thermodilution has been validated,^99^, ^100^ which allows direct measurement of absolute coronary blood flow and resistance. A specialized monorail infusion catheter is inserted over the pressure wire with its tip being positioned in the proximal region of the coronary artery. Then, room‐temperature saline is injected at a rate of 15−25 mL/min. After a steady‐state maximum hyperemia is induced, the distal temperature can be recorded by the guidewire. By pulling back the temperature sensor to the opening of the infusion catheter, the infusion temperature can be determined. Absolute blood flow and vessel resistance are calculated automatically using these variables. It has been well verified that hyperemic flow measured by continuous thermodilution correlates well with the gold standard PET.^101^

Microvascular resistance reserve (MRR) is a novel metric specific for the microvasculature, defined as the ratio of true resting to HMR.^102^ It is calculated as follows: MRR = (*Q*_max_/*Q*_rest_) × (*P*_a,rest_/*P*_d,hyper_), where *P*_a,rest_ represents aortic pressure at rest and *P*_d,hyper_ indicates distal coronary pressure measured at hyperemia, while *Q*_rest_ and *Q*_max_ denote the actually measured resting and hyperemic blood flow. MRR can be easily measured invasively using intracoronary Doppler, continuous thermodilution, or bolus thermodilution. However, Doppler tracing was shown to be challenging in obtaining high quality signals, resulting in insufficient data in up to 30% of patients,^103^ whereas bolus thermodilution is susceptible to patient‐ and operator‐dependent variability. The correlation between continuous thermodilution MRR and patient symptoms has been demonstrated, suggesting that it could be a superior technique to bolus thermodilution for reflecting the disease status of patients with CMVD.^104^

The optimal cutoff values of MRR remain to be determined. An exploratory analysis in patients with angina and nonobstructive CAD indicated that an MRR value of >2.7 ruled out CMVD with high certainty, whereas an MRR value < 2.1 highly suggested CMVD.^105^ The ILIAS registry study proposed MRR of 3 as the cutoff value to predict MACE and target vessel failure at 5‐year follow‐up in vessels with functionally significant epicardial disease.^106^ This unique metric seems useful in the occasion with adaptive coronary flow regulation such as AS.^107^ Measurement of MRR is accurate, reproducible, and safe, and is independent of epicardial coronary disease, hemodynamic variation, operator, autoregulation, epicardial resistance, and myocardial mass. With wider clinical applications, MRR will gain further clinical benefits.

#### 4.3.3 Intracoronary Doppler flow velocity measurement

This technique uses Doppler flow velocity recorder connected with a Doppler flow wire, which is inserted into a distal coronary artery to record the flow spectrum. It is followed by intracoronary adenosine injection to measure the coronary flow velocity during maximal hyperemia.^108^ The CFR value is obtained by calculating the ratio of diastolic coronary flow velocity in the maximal hyperemia to that in the basal state. The major advantage of this technique is that it allows accurate measurement of flow velocity and CFR in each coronary artery. The disadvantage is that flow velocity is affected by the guidewire position in the lumen, coronary flow velocity profile, and luminal area changes following injection of vasodilators. In addition, a reduced CFR can be observed in patients with CMVD or severe epicardial coronary stenosis and thus, coronary microvascular function can only be evaluated in patients without obstructive epicardial coronary stenosis. The normal CFR value is >2.5^65^, ^109^, ^110^ and CFR < 2.0 indicates the presence of coronary microvascular dysfunction.

HMR is a recently proposed index. This new technique uses an intracoronary guidewire equipped with the Doppler transducer and pressure sensor to measure the mean flow velocity and pressure in the cardiac cycle during maximal hyperemia in the distal end of a stenotic lesion (or distal coronary arteries in the absence of a stenotic lesion). The HMR value is calculated as follows: HMR = pressure/flow velocity.^111^ CFR < 2.5 and HMR > 1.7 mmHg/cm/s indicate coronary microvascular dysfunction. HMR is not affected by resting coronary blood flow, but the diagnostic cutoff value remains controversial.

**FIGURE 2:**
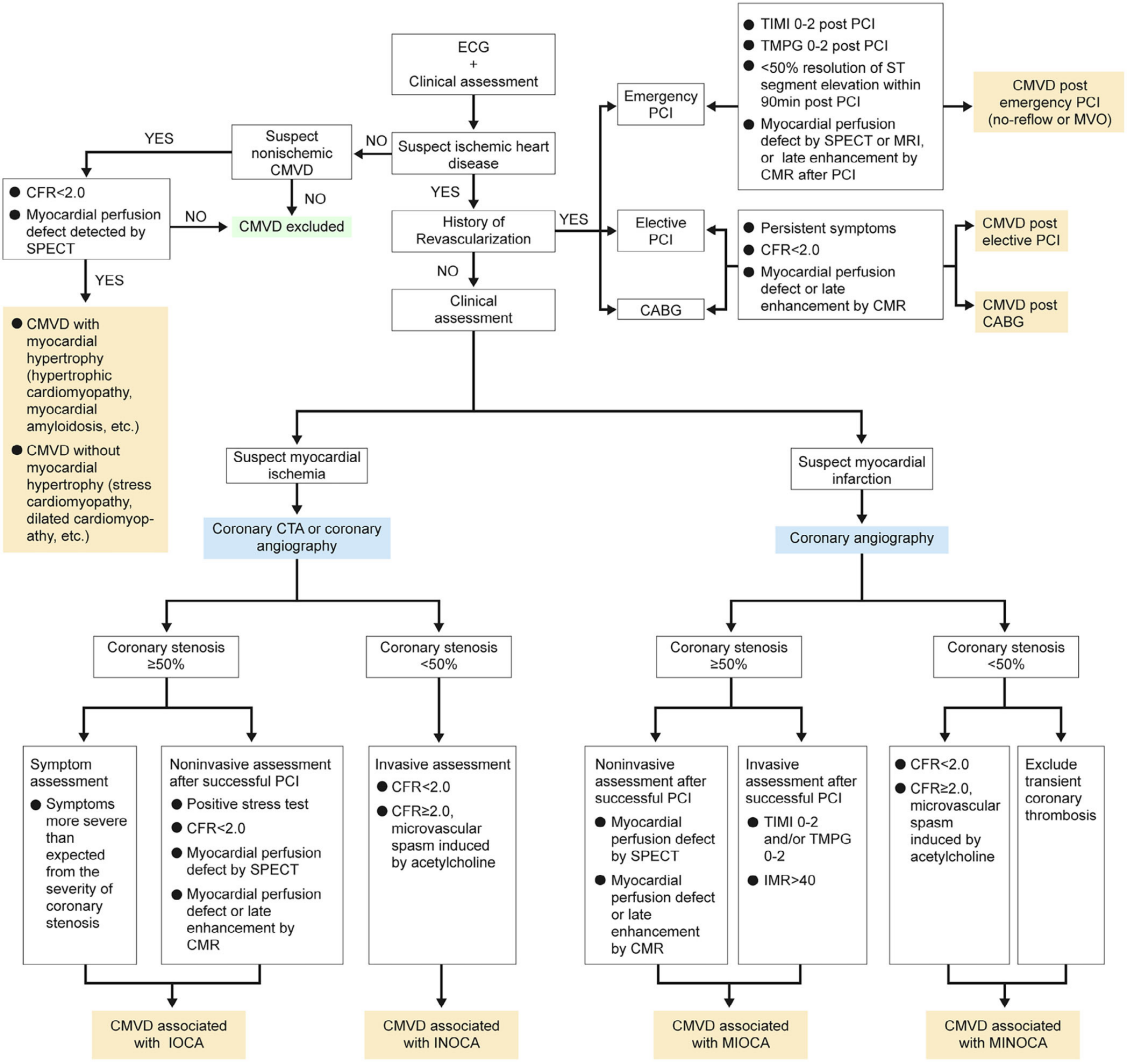
Diagram of the diagnostic flow for CMVD. CABG, coronary artery bypass grafting; CFR, coronary flow reserve; CMR, cardiovascular magnetic resonance; CMVD, coronary microvascular disease; CTA, computed tomography angiography; IMR, index of microcirculatory resistance; INOCA, ischemia with nonobstructive coronary artery; IOCA, ischemia with obstructive coronary artery; MINOCA, myocardial infarction with nonobstructive coronary arteries; MIOCA, myocardial infarction with obstructive coronary arteries; MVO, microvascular obstruction; MRI, magnetic resonance imaging; PCI, percutaneous coronary intervention; SPECT, single‐photon emission computed tomography; TIMI, thrombolysis in myocardial infarction; TMPG, TIMI myocardial blush grading. The figure was original and created using PowerPoint software.

The advantages and disadvantages, level of evidence, and recommended grades of invasive techniques for evaluating coronary microvascular function are shown in Table 6. The clinical diagnostic flow chart of CMVD is shown in Figure 2.

**TABLE 6:** Invasive techniques for evaluating coronary microvascular function.

Technique	Method	Drug load	Parameter	Diagnostic thresholds	Advantages	Limitations	Clinical significance	Class of recommendation	Level of evidence	References
Coronary angiography	Dynamic changes in the coronary artery filling with angiographic contrast using X‐ray photography/TIMI frames	Iodine contrast media	TIMI blood flow grade, TFC	TIMI grading ≤ 2, TFC > 25 fps	• Simple, feasible and inexpensive • Identification of epicardial coronary artery spasm and microvascular spasm after acetylcholine administration	• Only semi‐quantitative evaluation • Low sensitivity for CMVD diagnosis • Cannot reflect CFR Only used for resting state evaluation	Is a semi‐quantitative index to reflect the perfusion status of the coronary microcirculation	I	A	^86^, ^87^, ^89^, ^90^, ^91^
Intracoronary temperature‐pressure measurement	Estimation of coronary blood flow using bolus injection to calculate mean transit time or using continuous thermal dilution technique	Vasodilators: • Adenosine • Saline solution	CFR, IMR, MRR	CFR < 2–2.5, IMR > 25 U, MRR < 2.1	• CFR and IMR can be combined to assess both impaired dilation and enhanced contraction of microvessels • IMR specifically assesses microvascular function independent of resting‐state hemodynamics • MRR is accurate, reproducible, safe and independent of the operator, the autoregulation, the epicardial resistance, and the myocardial mass	• CFR does not distinguish between CMVD and epicardial coronary lesions • IMR cutoff value is still controversial • IMR correlates poorly with PET versus HMR • High intra‐ and inter‐observer variability	Can be used in a variety of clinical scenarios: post‐PCI, post‐STEMI, angina pectoris, myocardial ischemia with nonobstructive coronary arteries cardiomyopathy	IIa	B	^97^, ^98^, ^100^, ^101^, ^102^ ^,^ ^105^, ^106^, ^108^
Intracoronary Doppler flow velocity measurement	Recording peak coronary flow velocity using Doppler technique	Vasodilator: Adenosine	CFR, HMR	CFR < 2.5, HMR > 1.7 mmHg/cm/s	• CFR and HMR can be combined to assess both impaired dilation and enhanced contraction of microvessels • HMR independent of resting‐state coronary blood flow • Simultaneous measurement of FFR is possible • Good correlation with clinical prognosis and measurements by noninvasive techniques • Good reproducibility	• Complex technique • CFR unable to distinguish between CMVD and epicardial coronary lesions	For assessment of microvascular function caused by suspected CMVD or post myocardial infarction	IIb	C	^97^, ^98^, ^109^, ^110^, ^111^
Coronary spasm induction test	Assessment of epicardial coronary and microvascular spasm using intracoronary infusion of vasoactive drugs	Vasoactive drugs: • Acetylcholine • Ergonovine	Angina pectoris symptoms, Electrocardiographic changes, Changes in the internal diameter of blood vessels	• Epicardial coronary artery spasm: >90% reduction in the internal diameter of the vessel; • Microvascular spasm: Presence of angina and ECG ischemic changes without change in large vessel internal diameter	• Simple, easy to perform, and inexpensive • Can distinguish between epicardial coronary spasm and coronary microvascular spasm	• Additional contrast injection and radiation exposure • Inability to provide direct evidence of microcirculatory spasm • Risk of severe ventricular arrhythmias • Less clinical use	For suspected coronary spasm angina	IIb	B	^65^

Abbreviations: CMVD, coronary microvascular disease; CFR, coronary flow reserve; FFR, fractional flow reserve; HMR, hyperemic microvascular resistance; IMR, index of microcirculatory resistance; MRR, microvascular resistance reserve; PCI, percutaneous coronary intervention; PET, positron emission tomography; STEMI, ST‐segment elevation myocardial infarction; TFC, TIMI frame count; TIMI, thrombolysis in myocardial infarction.

## 5 CLINICAL MANIFESTATIONS AND DIAGNOSTIC CRITERIA OF CMVD

### 5.1 CMVD in patients with myocardial ischemia

#### 5.1.1 CMVD associated with INOCA

This type of CMVD is also referred to as primary stable MVA,^4^ where patients exhibit symptoms of persistent myocardial ischemia with a low CFR or laboratory evidence of microvascular spasm without obstructive lesions of the epicardial coronary artery. The primary symptom is exertional chest pain, which is difficult to distinguish from chest pain in patients with severe coronary stenosis. The following characteristics suggest the possibility of CMVD: Women are common with the majority of cases occurring after menopause; Most patients experience labor‐induced chest pain, and very few have chest pain during rest; A single episode of chest pain lasts for a relatively long time with more than 50% episodes lasting for more than 10 min, and the discomfort continues for several minutes after ceasing exercise; Nitroglycerin is ineffective against chest pain, or even make chest pain worse.

The following criteria are recommended for the diagnosis of CMVD associated with INOCA: (1) typical clinical symptoms; (2) at least one of the following objective evidence of myocardial ischemia: exertion‐induced or spontaneous typical chest pain accompanied by ST‐segment depression on ECG; reversible myocardial perfusion defect by SPECT; CFR reduction (<2.0) during stress by Doppler ultrasound or MPRI reduction (<2.0) by CMR; and metabolic evidence of myocardial ischemia by PET (3) CAG shows normal coronary artery, or irregular coronary walls or a luminal stenosis < 50%^109^; (4) If CMVD is highly suspected clinically but CFR is ≥2.0, intracoronary acetylcholine provocation test can be used under close supervision. The diagnosis of CMVD is confirmed if angina symptoms and ischemic ST‐T changes on ECG appear without spasm in the epicardial coronary arteries; (5) noncardiac chest pain and other cardiac conditions, such as variant angina pectoris, cardiomyopathy, myocarditis, or valve heart disease should be ruled out.

#### 5.1.2 CMVD associated with IOCA

When stable angina is caused by combined CMVD and epicardial coronary obstructive lesions, patients may experience a prolonged angina attack with a high variability in the threshold of physical activity triggering angina and an ineffective response of sublingual nitroglycerin. In addition, the severity of angina often exceeds that expected from the degree of coronary stenosis.

The following criteria are recommended for the diagnosis of CMVD associated with IOCA: (1) typical clinical symptoms; (2) a positive stress test early after a successful PCI; (3) CFR < 2.0, or a positive intracoronary acetylcholine provocation test that induces typical angina pectoris and ischemic ST‐T changes without visible epicardial coronary artery spasm after coronary stenosis is relieved by PCI; (4) TIMI flow grade < 3 and/or TMPG < 3 in patients receiving elective PCI; (5) myocardial perfusion defect detected by SPECT or MRI, or late gadolinium enhancement displayed by MRI in patients receiving PCI before discharge. However, in post‐PCI patients, whether PCI‐related CMVD exists requires further clarification.

### 5.2 CMVD in patients with MI

#### 5.2.1 CMVD associated with MINOCA

MINOCA is a syndrome with symptoms of non‐STEMI and laboratory evidence of coronary microvascular dysfunction after excluding epicardial obstructive and spastic coronary lesions, transient coronary thrombosis, cardiomyopathy, and other cardiovascular diseases. The most frequent causes of MINOCA are plaque rupture or erosion, thromboembolism, hereditary thrombophilia, or MVO associated with microvascular spasm.^6^, ^120^ The clinical presentation is recurrent chest pain during rest or in the early morning, and chest pain induced by mild physical activity, which may last for 1–2 h, and nitroglycerin administration is ineffective. Ischemic and dynamic ST‐T changes on ECG can be recorded during the onset of chest pain or on Holter monitoring. Patients with MINOCA have significantly higher MACE and lower quality of life, but the clinical diagnostic pathway is to be defined.

#### 5.2.2 CMVD associated with MIOCA

This type of CMVD is frequently linked to MVO. The incidence of post‐PCI left ventricular remodeling, heart failure, and mortality are higher in patients with STEMI and MVO.^121^, ^122^ One year follow‐up after successful PCI found that one‐third to one‐half of patients experienced CMVD‐related angina with left ventricular remodeling, cardiac dysfunction and cardiovascular events.^123^, ^124^ Endothelial dysfunction, oxidative stress, decreased NO generation, prior CMVD, and ischemia/reperfusion injury have been found to be contributing factors to CMVD in MVO. However, it is necessary to take into account of the possibility of emergency PCI‐related CMVD.

### 5.3 CMVD associated with revascularization

#### 5.3.1 CMVD associated with emergency PCI

The incidence of MVO is 5–50% in patients with STEMI receiving direct PCI. The following conditions indicate the existence of MVO: (1) TIMI blood flow grades 0–2 after PCI; (2) TMPG classes 0–2 after PCI; (3) ST‐segment elevation on ECG resolves by <50% 90 min after PCI; (4) SPECT shows regional myocardial no‐perfusion area before discharge, and MRI shows a first‐pass perfusion defect, or late gadolinium   enhancement. In addition, in patients receiving PCI due to acute coronary syndrome (ACS) or restenosis after great saphenous vein bypass grafting, atheromatous debris from the compressed plaque may go to the distal vessel and cause microembolization and small MI.

#### 5.3.2 CMVD associated with elective PCI

About one‐third of patients receiving elective PCI can develop CMVD, which is manifested by increased troponin levels after PCI, recurrent angina pectoris, and an increased risk of major cardiovascular events, death, MI, and repeated PCI. The mechanism involves the distal embolism of plaque materials during stent implantation, preexistence of CMVD before PCI, an increased α‐adrenergic sympathetic nerve tension in coronary microvessels caused by balloon dilation, and the aggravation of the original endothelial dysfunction caused by stent‐eluted drugs.^125^, ^126^ The diagnostic criteria of elective PCI‐related CMVD include: (1) typical clinical manifestation; (2) CFR < 2.5 measured by intracoronary Doppler ultrasound post‐PCI^127^; (3) IMR ≥ 25 measured immediately post‐PCI.^21^

#### 5.3.3 CMVD associated with CABG

If angina pectoris occurs repeatedly after CABG, the combined CMVD should be considered in most cases, and the mechanism involves structural and functional abnormalities of coronary microvessels.^60^, ^126^, ^128^ During CABG, many factors may affect the function of coronary microvessels, including cardiac arrest, extracorporeal circulation, myocardial ischemia, and inflammatory response. The effect of myocardial injury after CABG on the prognosis of patients is similar to that of PCI, indicating that regardless of the mechanism, prognosis ultimately depends on the extent of myocardial necrosis.^128^ Myocardial injury post‐CABG may be related to CMVD‐induced electrical instability or sustained myocardial ischemia. In addition, CMVD is also common in allograft coronary angiopathy in heart transplant recipients, which is independent from epicardial coronary angiopathy and associated with the risk of death.

### 5.4 CMVD associated with non‐atherosclerotic heart disease

#### 5.4.1 CMVD with myocardial hypertrophy

*CMVD associated with HCM*: Microvascular pathological features include arteriolar wall thickening, luminal narrowing, and capillary rarefaction. Many studies have shown that although CFR reduction is detected in non‐hypertrophic myocardial areas, it is more significant in subendocardial and hypertrophic areas. Long‐term CMVD may induce recurrent myocardial ischemia and myocardial cell death, leading to local myocardial fibrosis. In patients with HCM, CMR shows late gadolinium enhancement image. CMVD detected by PET is a reliable predictor of left ventricular remodeling, systolic dysfunction, clinical deterioration and death.^42^

*CMVD associated with Anderson–Fabry disease*: CMVD has become an important feature of Anderson–Fabry disease‐related cardiomyopathy. A considerable number of patients with this disease experience angina pectoris without coronary artery stenosis. Recent studies have shown that mild coronary microvascular dysfunction is the phenotype characteristics before the occurrence of myocardial hypertrophy.

*CMVD associated with cardiac amyloidosis*: The pathogenesis includes arteriolar wall infiltration and thickening, luminal stenosis, microvascular dysfunction caused by autonomic nervous dysregulation and endothelial dysfunction, extramural compression by interstitial deposition of amyloid protein, and reduced microvascular perfusion pressure caused by elevated left ventricular filling pressure. In patients with systemic amyloidosis, angina pectoris with decreased CFR may occur before typical general manifestations.^26^

*CMVD associated with AS*: About 40% of patients with AS develop angina pectoris without epicardial CAD, which increases the risk of sudden death. These patients exhibit decreased MBF and CFR and weakened exercise tolerance. Reduced CFR is the only independent predictor of cardiovascular events in patients with AS.^129^ Thanscatheter and transthoracic aortic valve replacement can restore myocardial perfusion and contractility by reducing left ventricular wall stress, and improve coronary microvascular function.^130^

#### 5.4.2 CMVD without myocardial hypertrophy

*Stress cardiomyopathy (Takotsubo cardiomyopathy)*: A study has shown that CMVD may be involved in the pathogenesis of Takotsubo cardiomyopathy.^131^ Transthoracic Doppler ultrasound or PET showed decreased coronary microvascular blood flow and CFR during the acute phase of Takotsubo cardiomyopathy. However, CMVD was reversible in most patients. In another study, the perfusion defects observed in myocardial segments with weakened systolic function were improved after intracoronary injection of adenosine and completely resolved after 1 month of follow‐up.^132^ The long‐term prognosis of patients showing slow blood flow on CAG is poor.^28^

*Dilated cardiomyopathy*: Recent studies have found that myocardial ischemia caused by CMVD is an independent risk factor of the progression of dilated cardiomyopathy. Patients with dilated cardiomyopathy and moderate or severe left ventricular remodeling often have abnormal myocardial perfusion. In addition, the severity of CMVD is an independent predictor of the risk of death and aggravated heart failure in patients with dilated cardiomyopathy.^133^

*HFpEF*: HFpEF has been redefined as a systemic disease characterized by multiple organ inflammation and microvascular dysfunction. CMVD plays a key role in the pathogenesis and progression of HFpEF.^134^ In a small, prospective observational study, mean CFR in a group of patients with HFpEF was significantly reduced and mean IMR was substantially increased compared with the control group. In addition, more than one‐third of these patients presented with evident CMVD, which was associated with death or hospitalization due to heart failure during follow‐up.^135^, ^136^ In patients with HFpEF, coronary artery hemodynamics measured with Doppler guidewire demonstrated that CMVD was mainly caused by endothelium‐dependent and endothelium‐independent microvascular dysfunction.^137^ Female patients with CMVD often have an impaired left ventricular diastolic function, and CMVD increases the risk of HFpEF.^138^ However, the causal relationship between CMVD and HFpEF is still unclear.

*Diabetic cardiomyopathy*: Recent prospective studies suggested that diabetic cardiomyopathy may be a unique and high‐risk clinical phenotype of DM‐related HFpEF, which is characterized by increased serum levels of N‐terminal pro‐B‐type natriuretic peptide, myocardial fibrosis, vascular endothelial dysfunction, and increased incidence and mortality of heart failure.^139^ The mechanism of microvascular disease in diabetic patients involves deposition of advanced glycation end products, vascular inflammation, reduced NO production, and endothelial cell apoptosis, leading to microvascular dysfunction and capillary rarefaction.

## 6 TREATMENT OF CMVD

To date, a number of small randomized clinical trials or nonrandomized observational studies have reported cardiovascular outcomes in patients with CMVD.^140^, ^141^ Large‐sample randomized clinical trials with cardiovascular events as endpoints are underway. Different from the 2017 Chinese expert consensus, the current consensus recommended treatment strategies of CMVD based on evidence from the latest clinical outcome studies, and presented classes for recommendations and levels of evidence.

### 6.1 Treatment of CMVD associated with INOCA

#### 6.1.1 Risk factor management

Atherosclerosis is the pathological basis for CMVD in most patients, and traditional risk factors of atherosclerosis such as smoking, hypertension, hyperlipidemia, and diabetes may promote development of CMVD. Thus, primary prevention of atherosclerosis for controlling risk factors may help alleviate CMVD and symptoms of angina.

(1) *Hypertension*: Angiotensin‐converting enzyme inhibitors (ACEIs) or angiotensin receptor blockers (ARBs) are treatment of choice. Studies have shown that ACEI treatment improves angina symptoms and CFR in women with INOCA and hypertension, which is consistent with the overall WISE hypothesis.^142^ Another randomized clinical study found that enalapril increases CFR in patients with diabetes and hypertension.^143^ A meta‐analysis showed that therapy with ACEIs and ARBs is associated with significantly improved CFR in patients with hypertension and CMVD.^144^

(2) *Hyperlipidemia*: Several small‐sample studies have shown that statins significantly improve exercise tolerance, CFR, exercise‐induced tissue hypoperfusion, and quality of life in patients with INOCA.^144^, ^145^, ^146^, ^147^ The proprotein convertase subtilisin kexin type 9 (PCSK9) inhibitors evolocumab and alirocumab significantly improve vascular endothelial function, inhibit inflammation and oxidative stress, and stabilize atherosclerotic plaques while reducing low‐density lipoprotein cholesterol levels.^5^, ^60^, ^148^, ^149^ However, EVOCATION study found that evolocumab did not prevent microvascular dysfunction in patients undergoing PCI.^150^

(3) *Diabetes*: A study has shown that oral hypoglycemic drugs or insulin can improve coronary microvascular endothelial function.^151^ Metformin reduces body weight and insulin resistance, improves acetylcholine‐mediated endothelium‐dependent microvascular function, and alleviates ST‐segment depression and angina symptoms.^152^, ^153^ Sodium‐dependent glucose transporters 2 (SGLT2) inhibitors attenuate CMVD by improving vascular endothelial function and inhibiting smooth muscle cell proliferation, endothelial cell oxidative stress, and inflammatory responses.^151^, ^154^, ^155^

(4) *Microembolism*: Low‐dose aspirin can reduce microembolism after PCI.^156^, ^157^ Tegregrel, a novel P2Y12 receptor inhibitor, can inhibit the degradation of adenosine and increase the level of adenosine, which may improve CMVD by dilating microvessels.^142^, ^158^

#### 6.1.2 Lifestyle modifications

Lifestyle management in patients with CMVD is similar to that in those with atherosclerosis and includes healthy diet, smoking cessation, and weight control.^141^, ^159^ Individualized exercise programs and cardiac rehabilitation can improve angina symptoms, exercise tolerance, quality of life, and CFR in patients with CMVD.^160^, ^161^, ^162^ As coronary macrovascular and microvascular spasms are often induced by stress, especially in female patients, prevention from stress and psychological counseling may be necessary, and behavioral therapy may help relieve stress and reduce spastic angina.^2^, ^163^

#### 6.1.3 Stratified treatment for CMVD

Based on the results of the CorMicA trial, coronary function test with invasive pressure guidewire, and endothelial function test with acetylcholine stress are recommended for patients with INOCA to differentiate MVA from vasospastic angina (VSA). The diagnostic criteria for MVA are FFR > 0.8, CFR < 2.0, IMR ≥ 25, and HMR ≥ 1.9 as measured by a coronary pressure guidewire, and occurrence of angina symptoms and ischemic ST‐segment changes on ECG with epicardial coronary diameter constriction < 90% during acetylcholine testing. The diagnostic criteria for VSA are FFR > 0.8, CFR ≥ 2.0, IMR < 25, and HMR < 1.9 as measured by a coronary pressure guidewire, and occurrence of angina symptoms, and ischemic ST‐segment changes on ECG with coronary diameter constriction ≥90% during acetylcholine testing. The diagnostic criteria for combined MVA and VSA are FFR > 0.8, CFR < 2.0, IMR ≥ 25, and HMR ≥ 1.9 as measured by a coronary pressure guidewire, and occurrence of angina symptoms, and ischemic ST‐segment changes on ECG with coronary diameter constriction ≥90% during acetylcholine testing.^140^

*Treatment of MVA*. (1) *β‐Blockers*: Several studies found that nebivolol, a highly selective inhibitor of β1‐receptor, alleviates angina symptoms via increasing NO production and coronary artery dilation. Intracoronary injection of nebivolol increases CFR and reduces myocardial oxygen consumption. Carvedilol blocks both α‐ and β‐receptors and has been proven beneficial for CMVD.^164^, ^165^

(2) *Calcium channel blockers (CCBs)*: Mibefradil as both L‐type and T‐type CCB, reduces the frequency of angina in patients with coronary slow flow.^166^, ^167^ Amlodipine and benidipine as long‐acting dihydropyridine agents relieve symptoms of MVA patients.^168^, ^169^, ^170^

(3) *Nicorandil*: Nicorandil is an ATP‐sensitive potassium channel opener, which effectively dilates coronary microvessels and produces salutary effects on MVA. Available studies have shown that nicorandil improves microvascular function in patients with stable CHD and significantly lowers the incidence of adverse cardiovascular events in patients with MVA.^171^, ^172^

(4) *Ranolazine*: Ranolazine can inhibit Na^+^ inward flow, promote Ca^+^ outward flow to reduce intracellular calcium overload, and dilate coronary arteries to relieve MVA. An open‐label, multicenter trial found that ranolazine significantly reduced angina symptoms and increased exercise tolerance in patients with MVA.^173^

(5) *Trimetazidine*: A study has shown that trimetazidine increases exercise tolerance and significantly reduces the duration of ST depression in patients with INOCA and CMVD.^174^

(6) *ACEIs/ARBs*: ACEIs/ARBs increase CFR by improving microvascular remodeling and dysfunction and decrease cardiac load. Studies have demonstrated that ACEIs/ARBs alleviate angina symptoms in patients with CMVD by increasing CFR, reducing cardiac load, and improving microvascular remodeling.^175^, ^176^, ^177^

(7) *Ivabradine*: Ivabradine is a specific inhibitor of cardiac pacing current, which acts as an anti‐MVA agent by slowing heart rate and reducing myocardial oxygen consumption. Clinical studies demonstrated that ivabradine reduced MVA symptoms in patients with INOCA.^178^

(8) *Endothelin receptor antagonists*: Increased serum endothelin‐1 (ET‐1) levels in patients with MVA lowers the exercise threshold to trigger angina and reduces CFR in women. The CorMicA trial found that patients with INOCA showed enhanced response to ET‐1. In a randomized controlled study, the endothelin receptor antagonist zibotentan improved vascular endothelial function in patients with CMVD.^179^

(9) *Tricyclic antidepressants*: In patients with refractory angina despite routine therapies, low‐dose tricyclic antidepressants, such as amitriptyline, imipramine, or desmethylimipramine, might be considered as an alternative therapy.^142^, ^180^, ^181^

(10) *Nonpharmacological treatment*: In patients with refractory MVA and failed medical treatment, spinal cord electrical stimulation (SCS) or enhanced external counter pulsation (EECP) may be considered.^163^ Studies have shown that SCS reduces MVA frequency and duration, and improves Seattle Angina Questionnaire scores and quality of life in patients with CMVD.^163^, ^182^, ^183^ EECP relieves MVA by increasing retrograde aortic blood flow, prolonging diastolic period, and augmenting coronary perfusion, venous return flow, and cardiac output.^184^

*Treatment of VSA*. (1) CCBs: CCBs are the drug of choice for the treatment of VSA, and the dose of CCBs should be doubled in patients with severe coronary spasms. Combined administration of dihydropyridine and non‐dihydropyridine CCBs, such as diltiazem, is necessary in special cases.^185^, ^186^ Benidipine, a controlled‐release dihydropyridine CCB, reduces the symptoms and improves the prognosis of patients with VSA.^168^, ^187^

(2) *Nitrates*: Long‐acting nitrates alone or in combination with CCBs can reduce the frequency of angina in patients with VSA.^185^, ^188^, ^189^, ^190^

(3) *Nicorandil*: Nicorandil improves VSA by opening ATP‐sensitive potassium channels and dilating microvessels.^7^, ^191^, ^192^

(4) *Rho kinase inhibitors*: Fasudil, a Rho kinase inhibitor, inhibits acetylcholine‐induced microvasospasm and alleviates VSA. In patients with VSA and elevated IMR, fasudil significantly decreases IMR.^193^, ^194^

(5) *Phosphodiesterase (PDE) inhibitors*: Cilostazol, a PDE3 inhibitor, inhibits adenosine degradation and exerts antiplatelet, anti‐inflammatory, and vasodilatation effects. Cilostazol alleviates spontaneous or ergometrine‐induced VSA and refractory angina unresponsive to calcium antagonists and nitrates.^195^ Sildenafil, a PDE5 inhibitor, dilates coronary arteries and improves endothelium‐dependent vasodilation in patients with CHD. Moreover, sildenafil improves CFR and relieves angina symptoms in female patients with INOCA whose CFR is <2.5.^196^

(6) *β‐Blockers*: In patients with VSA, β‐blockers, especially nonselective β‐blockers such as propranolol, should be avoided.^197^

*Treatment of combined MVA and VSA*. Statins improve vascular endothelial function and inhibit vascular inflammation.^7^ Recommendations for treatment of CMVD in INOCA are shown in Table 7.

**TABLE 7:** Recommended treatment of CMVD associated with INOCA.

Treatment	Class of recommendation	Level of evidence	References
Risk factor management			
Hypertension			
ACEIs/ARBs	I	A	^142^, ^143^, ^144^
Hypercholesterolemia			
Statins	I	A	^60^, ^144^, ^146^, ^147^
PCSK9 inhibitors	IIb	B	^60^, ^148^
Diabetes mellitus			
Metformin	IIa	B	^152^, ^153^
SGLT2 inhibitors dapagliflozin and empagliflozin	IIa	B	^154^, ^155^
Thromboembolism			
Low‐dose aspirin	IIa	B	^156^, ^157^
Clopidogrel or ticagrelor in patients with aspirin intolerance	IIb	C	^158^
Lifestyle modifications			
Smoking cessation, body weight control, exercise, cardiac rehabilitation, stress avoidance	I	A	^141^, ^159^, ^160^, ^161^, ^162^, ^163^
Stratified therapy for CMVD			
Based on coronary function tests with an invasive pressure guidewire and endothelial function tests with acetylcholine injection to stratify diagnosis and treatment	IIa	B	^140^, ^145^
Treatment of MVA			
β‐Blockers such as nebivolol (not available in China)	I	A	^164^, ^165^
CCBs such as amlodipine or benidipine	I	B	^168^, ^169^, ^170^
Nicorandil	IIa	B	^171^, ^172^
Ranolazine	IIa	B	^173^
Trimetazidine	IIa	B	^174^
ACEIs /ARBs	IIa	B	^175^, ^176^, ^177^
Ivabradine	IIa	B	^178^
Endothelin receptor antagonists such as zibotentan (not available in China)	IIb	B	^179^
Imipramine (for patients less effective with conventional antianginal drugs or overreaction to cardiac pain)	IIb	B	^180^, ^181^
Spinal cord electrical stimulation or enhanced external counter pulsation (for patients with refractory MVA)	IIb	B	^163^, ^182^, ^183^, ^184^
Treatment of VSA			
Amlodipine or diltiazem	IIa	B	^168^, ^185^, ^186^
Nitrates	IIa	B	^185^, ^188^, ^189^, ^190^
Nicorandil	IIa	C	^192^
Fasudil, cilostazol, sildenafil	IIb	B	^193^, ^194^, ^195^, ^196^
Propranolol	III	C	^197^
Treatment of MVA combined with VSA			
CCBs such as amlodipine, diltiazem, or verapamil	IIa	B	^168^, ^169^, ^170^
ACEIs /ARBs	IIa	B	^143^, ^144^
Nicorandil	IIa	B	^192^
Trimetazidine	IIa	B	^174^
Statins	IIa	C	^144^, ^146^, ^147^

Abbreviations: ACEIs, angiotensin‐converting enzyme inhibitors; ARBs, angiotensin receptor blockers; CCB, calcium channel blocker; CMVD, coronary microvascular disease; INOCA, ischemia with nonobstructive coronary arteries; MVA, microvascular angina; PSCK9, proprotein convertase subtilisin kexin type 9; SGLT2, sodium‐dependent glucose transporters 2; VSA, vasospastic angina.

### 6.2 Treatment of CMVD associated with IOCA

#### 6.2.1 Lifestyle modifications

Improvements in lifestyle, such as healthy diet, smoking cessation, physical exercise, cardiac rehabilitation, body weight control, stress avoidance, and psychological counseling, can improve the prognosis and CFR in patients with IOCA and CMVD.^163^, ^198^, ^199^, ^200^ Recommendations are shown in Table 8.

**TABLE 8:** Recommended treatment of CMVD associated with IOCA.

Treatment	Class of recommendation	Level of evidence	References
Lifestyle modifications			
Smoking cessation, body weight control, exercise, cardiac rehabilitation, stress avoidance	I	A	^163^, ^198^, ^199^, ^200^
Antiatherosclerosis therapy			
Antiplatelet therapy			
Low‐dose aspirin	IIa	B	^157^
Clopidogrel or ticagrelor in patients with aspirin intolerance	IIb	C	^207^
Ticagrelor instead of clopidogrel in patients at high risk of ischemia	IIb	C	^158^, ^207^
Secondary prevention of coronary heart disease			
Statins	I	A	^60^, ^144^, ^145^, ^201^, ^202^
ACEIs/ARBs	I	A	^143^, ^144^, ^203^, ^204^
β‐Blockers	I	A	^205^, ^206^
Stratified therapy for CMVD			
Based on coronary function tests with an invasive pressure guidewire and endothelial function tests with acetylcholine stress to stratify diagnosis and treatment	IIa	B	^140^, ^145^
Treatment of CMVD	Same as the treatment of CMVD in INOCA	Same as the treatment of CMVD in INOCA	Same as the treatment of CMVD in INOCA
Coronary revascularization			
Revascularization can be performed when a major coronary artery stenosis is ≥90% or in case of extensive myocardial ischemia or FFR < 0.8	I	A	^5^, ^208^

Abbreviations: ACEIs, angiotensin‐converting enzyme inhibitors; ARBs, angiotensin receptor blockers; CMVD, coronary microvascular disease; INOCA, ischemia with nonobstructive coronary arteries; IOCA, ischemia with obstructive coronary arteries; MVA, microvascular angina; VSA, vasospastic angina.

#### 6.2.2 Antiatherosclerosis therapy

Previous studies have shown that statins, ACEIs/ARBs, β‐blockers, and aspirin/P2Y12 receptor inhibitors can improve the prognosis and CFR in IOCA patients with CMVD.^5^, ^60^, ^142^, ^144^, ^145^, ^157^, ^201^, ^202^, ^203^, ^204^, ^205^, ^206^, ^207^ The recommendations are shown in Table 8.

#### 6.2.3 Stratified medical therapy for CMVD

Coronary function tests with an invasive pressure guidewire and endothelial function tests with acetylcholine stress are recommended to detect CFR and IMR, respectively, before and after revascularization and to stratify treatment for MVA and VSA. The treatment protocol is similar to the aforementioned treatment for patients with INOCA and CMVD^5^, ^7^, ^140^, ^145^ and shown in Table 8.

#### 6.2.4 Coronary revascularization

Multiple coronary revascularization guidelines recommend direct intervention when a major coronary artery stenosis is ≥90%, or coronary stenosis is <90% in combination with evidence of extensive myocardial ischemia or FFR < 0.8. In patients with IOCA and CMVD, epicardial artery revascularization can improve angina symptoms and prognosis.^5^, ^208^ The recommendations are shown in Table 8.

### 6.3 Treatment of CMVD associated with MINOCA

#### 6.3.1 Stratified medical therapy for CMVD

Classification, diagnosis, and treatment of CMVD according to the results of coronary artery function evaluation and acetylcholine‐mediated endothelial function assessment are recommended. The recommended treatment is the same as that for INOCA‐associated CMVD,^6^, ^140^, ^145^, ^209^ as shown in Table 9.

**TABLE 9:** Recommended treatment of CMVD associated with MINOCA.

Treatment	Class of recommendation	Level of evidence	References
Stratified medical therapy for CMVD			
Based on coronary function tests with an invasive pressure guidewire and endothelial function tests with acetylcholine injection to stratify diagnosis and treatment	IIa	B	^140^, ^145^
Treatment of CMVD	Same as the treatment of CMVD in INOCA	Same as the treatment of CMVD in INOCA	Same as the treatment of CMVD in INOCA
Secondary prevention of CHD			
Statins	I	A	^211^, ^212^, ^213^
ACEIs/ARBs	I	A	^211^, ^212^, ^213^
β‐blockers	I	A	^211^, ^212^, ^213^
Aspirin combined with clopidogrel or ticagrelor	IIb	A	^211^, ^212^, ^213^

Abbreviations: ACEIs, angiotensin‐converting enzyme inhibitors; ARBs, angiotensin receptor blockers; CHD, coronary heart disease; CMVD, coronary microvascular disease; INOCA, ischemia with nonobstructive coronary arteries; MINOCA, myocardial infarction with nonobstructive coronary arteries; MVA, microvascular angina; VSA, vasospastic angina.

#### 6.3.2 Secondary prevention of MINOCA

Current observational or prospective studies suggest that secondary prevention of CHD improves long‐term outcomes in patients with MINOCA. In the SWEDEHEART registry, 9136 patients with MINOCA received statins, ACEIs/ARBs, β‐blockers, and dual‐antiplatelet therapy, who were followed for an average of 4.1 years. The results showed that statins and β‐blockers significantly reduced the incidence of major cardiovascular events (all‐cause mortality, MI, ischemic stroke, and heart failure), while dual‐antiplatelet therapy did not reduce major cardiovascular events.^6^, ^210^ Choo et al.^211^ followed 396 patients with MINOCA for 2 years and found that ACEIs/ARBs and statins significantly reduced all‐cause mortality in patients with MINOCA. Paolisso et al.^212^ conducted a prospective study on 134 patients with MINOCA with an average follow‐up of 20 months and found that ACEIs/ARBs significantly reduced the incidence of all‐cause mortality and MACEs; however, dual‐antiplatelet therapy, β‐blockers, and statins did not produce significant benefits. Another retrospective study showed no benefits from aspirin therapy in patients with MINOCA due to coronary spasm.^213^ The ongoing PROMISE trail including 140 MINOCA patients aims to compare the precision medicine approach with standard medical treatment to improve the angina status and the rate of MACEs during 12‐month follow‐up.^214^ Therefore, statins, ACEIs/ARBs, and β‐blockers may improve the long‐term prognosis of patients with MINOCA, partly due to improved CMVD (Table 9).

### 6.4 Treatment of CMVD associated with MIOCA

Prevention of MVO and no‐reflow in patients with STEMI receiving primary PCI is a key for the prevention and treatment of CMVD.^215^

#### 6.4.1 Pharmacological treatment before PCI

(1) *Dual‐antiplatelet therapy*: This is the cornerstone of antithrombotic therapy for STEMI. Ticagrelor combined with aspirin is recommended in patients with STEMI who have a low risk of bleeding, and clopidogrel can be used in patients who are intolerant to ticagrelor or have a high risk of bleeding.^216^, ^217^, ^218^

(2) *Statins*: MCE studies have shown that statin therapy before revascularization in patients with ACS significantly improves coronary microvascular perfusion.^8^, ^219^, ^220^

#### 6.4.2 Pharmacological treatment during PCI

(1) *Platelet glycoprotein IIb/IIIa receptor antagonists (tirofiban, abciximab, or eptifibatide; the latter two not commercially available in China)*: In patients receiving primary PCI with a high thrombus burden, intracoronary or intravenous administration of platelet glycoprotein IIb/IIIa receptor antagonists reduces MVO incidence and MI size, improves myocardial perfusion, and lowers the reinfarction rate and mortality.^221^

(2) *Plasminogen activators*: Low‐dose streptokinase by intracoronary injection immediately after primary PCI improves myocardial reperfusion but not long‐term left ventricular size or function.^222^ The T‐TIME investigators found that in patients with STEMI, adjunctive low‐dose intracoronary alteplase during primary PCI does not reduce MVO.^223^

(3) *Adenosine*: The REOPEN‐AMI study showed that in patients with STEMI, intracoronary administration of adenosine after coronary thrombus aspiration improves MVO and left ventricular function and reduces rehospitalization.^224^

(4) *Nicorandil*: Studies have shown that intracoronary injection of nicorandil during PCI prevents or alleviates no‐reflow and improves myocardial perfusion and clinical prognosis.^225^ The Effects of Nicorandil Administration on Infarct Size in Patients With ST‐Segment‐Elevation Myocardial Infarction Undergoing Primary Percutaneous Coronary Intervention (CHANGE) study showed that perioperative intravenous administration of nicorandil in patients with STEMI undergoing primary PCI reduces the occurrence of coronary no‐reflow/slow flow and promotes ST‐segment restoration.^226^

(5) *Nitroprusside*: Studies have demonstrated that intracoronary administration of nitroprusside improves no‐reflow during primary PCI and increases myocardial perfusion.^227^, ^228^

(6) *Verapamil and diltiazem*: Both drugs reduce the occurrence of slow flow or no‐reflow by dilating epicardial coronary arteries and coronary arterioles.^229^

#### 6.4.3 Nonpharmacological treatment

(1) *Coronary thrombus aspiration*: Routine thrombus aspiration is not recommended. However, in cases with a high coronary thrombus burden, thrombus aspiration may be performed to reduce MVO and improve coronary microvascular function and myocardial reperfusion.^230^

(2) *Proximal or distal protection devices*: A randomized, prospective, multicenter clinical trial found that use of proximal and distal protection devices during saphenous vein graft intervention significantly reduces the incidence of perioperative MI.^231^

(3) *Excimer laser coronary atherectomy (ELCA)*: ELCA ablation can be considered in patients with a high intracoronary thrombus burden or undergoing saphenous vein graft intervention to reduce the occurrence of no‐reflow.^232^

(4) *Deferred stenting*: The DEFER‐STEMI study showed that after thrombus aspiration or balloon dilation, if TIMI flow was restored to grade 3, coronary stenting could be deferred until 4−16 h later. Compared with direct PCI, deferred stenting had a lower incidence of no‐reflow/slow reflow.^233^ The above treatment recommendations are listed in Table 10.

**TABLE 10:** Recommended treatment of CMVD associated with MIOCA.

Treatment	Class of recommendation	Level of evidence	References
Perioperative antiplatelet therapy			
Aspirin	I	A	^216^, ^217^
Ticagrelor or clopidogrel	I	A	^216^, ^218^
Statins	I	A	^219^, ^220^
Platelet glycoprotein IIb/IIIa receptor antagonists	IIa	B	^221^
Plasminogen activators	IIb	B	^222^
Adenosine	IIa	B	^224^
Nicorandil	IIa	B	^225^, ^226^
Nitroprusside	IIa	C	^227^, ^228^
Verapamil or diltiazem	IIa	C	^229^
Thrombus aspiration	IIa	B	^230^
Excimer laser coronary atherectomy	IIa	B	^232^
Proximal or distal protection devices	IIb	B	^231^
Deferred stenting	IIb	B	^233^

Abbreviations: CMVD, coronary microvascular disease; MIOCA, myocardial infarction with obstructive coronary arteries.

### 6.5 Treatment of CMVD after successful intervention for acute MI

Successful opening of infarct‐related artery in patients with STEMI is only the first step. The ultimate goal of treatment is to prevent CMVD, protect myocardial function, and improve patient prognosis.^121^, ^234^

#### 6.5.1 Drugs

(1) *β‐Blockers*: Studies have found that the third‐generation β‐blockers carvedilol and nebivolol improve microcirculatory perfusion and reduce infarct size in patients with STEMI. The Effect of Metoprolol in Cardioprotection During an Acute Myocardial Infarction (METOCARD‐CNIC) study showed that metoprolol significantly reduced myocardial infarct size at 6 months, improved LVEF, and significantly reduced the incidence of cardiovascular events at 2 years in patients with STEMI receiving intravenous metoprolol prior to PCI followed by oral metoprolol over 24 h. A CMR study revealed that metoprolol reduced MVO by 40% by a mechanism related to the inhibition of neutrophil activation.^235^, ^236^ In the EARLY‐BAM study,^237^ patients with STEMI who received intravenous metoprolol before primary PCI did not demonstrate significant reduction in myocardial infarct size at 30 days in the treatment group. However, recent international guidelines still recommend intravenous β‐blockers before PCI in the absence of heart failure and hypotension.^215^, ^238^

(2) *Statins*: The STATIN STEMI study found that high‐dose atorvastatin loading before primary PCI in patients with STEMI reduced the incidence of MVO but not myocardial infarct size.^239^ A subgroup analysis of SECURE‐PCI study showed that high‐dose atorvastatin administered pre‐ or 24 h postprimary PCI reduced the 30‐day cardiovascular event rate by 50%.^240^

(3) *Adenosine*: Studies have shown that continuous intravenous adenosine infusion during PCI significantly reduces the myocardial infarct size by 67% and improves coronary microcirculation in patients.^241^, ^242^, ^243^

(4) *Nicorandil*: Intracoronary injection of nicorandil during primary PCI improves myocardial perfusion, reduces myocardial infarct size, and ameliorates clinical prognosis.^225^ The CHANGE study showed that perioperative intravenous nicorandil administration for 24‐h in patients with STEMI significantly increased LVEF after a 6‐month follow‐up.^226^

(5) *Atrial natriuretic peptide* (ANP): A study has shown that ANP administration reduces occurrence of MVO by inhibiting ET‐1.^244^ The J‐WIND study found that ANP agonists administered before PCI in patients with STEMI significantly reduced the myocardial infarct size.^245^

(6) *Antiplatelet therapy*: Pre‐PCI administration of P2Y12 receptor inhibitors in patients with STEMI reduces MVO and infarct size and improves long‐term prognosis. In addition to inhibition of platelet aggregation, ticagrelor dilates microvessels and improves CMVD through the adenosine pathway, similar to postischemic conditioning.^158^ The On‐TIME‐2 study found that routine high‐dose tirofiban administration before PCI in patients with STEMI reduced microvascular injury, promoted ST‐segment regression, and improved clinical prognosis.^246^ The INFUSE‐AMI study showed that acute intracoronary administration of abciximab during PCI reduced the infarct size in patients with acute anterior MI.^247^

(7) *Erythropoietin* (EPO): In a Japanese study, intravenous EPO administration after PCI in patients with acute anterior MI reduced the incidence of transmural MI, significantly improved CFVR for 8 months, and reduced left atrial volume.^248^ However, more clinical studies are needed to confirm this therapeutic effect of EPO.

(8) *Iron chelators*: MVO and intramyocardial hemorrhage exacerbate iron deposition in the myocardial interstitium, which can induce an inflammatory response and exacerbate microvascular injury and left ventricular remodeling. Pre‐PCI administration of iron chelators in patients with STEMI significantly reduces serum iron and oxidative stress levels.^249^

#### 6.5.2 Nonpharmacological treatment

(1) *Ischemic conditioning*: Ischemic preconditioning refers to repeated coronary ballooning to block coronary arteries temporarily several times prior to coronary stenting to induce transient myocardial ischemia and protection against ischemia–reperfusion injury after PCI. Ischemic postconditioning refers to repeated coronary ballooning to block culprit coronary arteries temporarily several times after coronary stenting to induce transient myocardial ischemia and protection against ischemia–reperfusion injury after PCI. Studies have shown that ischemic postconditioning reduces MVO occurrence and promotes recovery of left ventricular function. Remote ischemic preconditioning and remote ischemic postconditioning involve the use of a cuff to inflate and deflate the upper limb several times before or after PCI in patients with STEMI, resulting in transient ischemia in the upper limb and local production of protective substances for myocardial ischemia, thereby promoting myocardial survival. The CONDI study, which included 166 patients with STEMI who received 5‐min upper arm inflation and deflation four times in a prehospital ambulance, showed that remote ischemic preconditioning reduced the myocardial infarct size but did not improve CBF. At the end of the 4‐year follow‐up, all‐cause mortality, cardiovascular events, and cerebrovascular events were significantly reduced in patients receiving remote ischemic preconditioning.^250^ White et al.^251^ found that remote ischemic preconditioning reduced myocardial infarct size and myocardial edema detected by CMR. However, the CONDI‐2/ERIC‐PPCI study found that remote ischemic conditioning offers no benefits on cardiovascular mortality and rehospitalization due to heart failure one year after primary PCI in patients with STEMI, and a CMR subgroup study showed that remote ischemic preconditioning did not reduce MI size or improved LVEF 6 months after PCI.^252^, ^253^

(2) *Pressure‐controlled intermittent coronary sinus occlusion (PICSO)*: PICSO improves coronary microcirculation and reduces the incidence of MVO during primary PCI by increasing the redistribution of MBF in ischemic regions, promoting the clearance of microvascular harmful substances, and inducing production of vascular growth factors.^254^The above treatment recommendations are summarized in Table 11.

**TABLE 11:** Recommended treatment of CMVD after successful PCI for acute myocardial infarction.

Treatment	Class of recommendation	Level of evidence	References
The ultimate goal of reperfusion therapy in patients with AMI is to attenuate CMVD and protect cardiac function	I	A	^121^, ^234^
β‐Blockers	I	A	^235^, ^236^, ^238^
Statins	I	A	^239^, ^240^
Adenosine	IIa	B	^241^, ^242^, ^243^
Nicorandil	IIa	B	^225^, ^226^
Platelet glycoprotein IIb/IIIa receptor antagonists	IIa	B	^246^, ^247^
Ticagrelor	IIb	B	^158^
ANP	IIb	B	^245^
Erythropoietin	IIb	B	^248^
Iron chelators	IIb	C	^249^
Ischemic conditioning	IIb	B	^250^, ^251^, ^252^, ^253^
PICSO	IIb	C	^254^

Abbreviations: AMI, acute myocardial infarction; ANP, atrial natriuretic peptide; CMVD, coronary microvascular disease; PCI, percutaneous coronary intervention; PICSO, pressure‐controlled intermittent coronary sinus occlusion.

### 6.6 Treatment of CMVD associated with non‐atherosclerotic heart disease

#### 6.6.1 Treatment of CMVD with myocardial hypertrophy

(1) *Treatment of primary disease*: In patients with hypertensive left ventricular hypertrophy, ACEIs/ARBs or CCBs may improve ventricular remodeling and increase CFR.^255^, ^256^, ^257^ In patients with hypertrophic obstructive cardiomyopathy, septal resection or chemical ablation may reduce left ventricular outflow tract obstruction and increase CFR.^258^, ^259^ In patients with severe AS, aortic valve replacement may reduce transvalvular pressure differences and increase coronary perfusion pressure, thereby improving CFR.^25^, ^260^, ^261^

(2) *Treatment of CMVD*: A meta‐analysis has shown that ACEIs and ARBs significantly improved the CFR in patients with hypertensive cardiac hypertrophy combined with CMVD.^144^ In patients with HCM, β‐blockers and CCBs increased CFR by reducing left ventricular end‐diastolic pressure and decreasing left ventricular outflow tract pressure differences.^262^, ^263^, ^264^, ^265^ Another study showed that β‐blockers significantly reduced the incidence of all‐cause mortality, cardiovascular death, and sudden cardiac death in patients with mild‐to‐moderate asymptomatic AS.^266^

#### 6.6.2 Treatment of CMVD without myocardial hypertrophy

ACEIs/ARBs are the first‐line drugs for the treatment of dilated cardiomyopathy combined with heart failure. Enalapril and quinapril improve CFR in patients with dilated cardiomyopathy.^142^, ^143^, ^267^ Other studies suggested that early administration of carvedilol in patients with idiopathic dilated cardiomyopathy significantly increased CFR which was closely associated with improved long‐term left ventricular function.^268^, ^269^ Nebivolol and esmolol increase CFR in patients with dilated cardiomyopathy.^270^, ^271^ Allopurinol significantly improves CFR and left ventricular systolic and diastolic functions while lowering serum uric acid levels in patients with dilated cardiomyopathy combined with hyperuricemia.^272^ Treatment recommendations are summarized in Table 12.

**TABLE 12:** Recommended treatment of CMVD associated with non‐atherosclerotic heart disease.

Treatment	Class of recommendation	Level of evidence	References
CMVD with myocardial hypertrophy			
Hypertensive myocardial hypertrophy			
ACEIs/ARBs	I	A	^255^, ^256^, ^257^
CCBs	IIa	B	^255^, ^256^
Hypertrophic cardiomyopathy			
Septal resection or chemical ablation	IIa	B	^258^, ^259^
Aortic stenosis			
Aortic valve replacement	I	A	^25^, ^260^, ^261^
CMVD treatment			
ACEIs/ARBs	I	A	^144^, ^255^, ^256^, ^257^
β‐Blockers	I	A	^264^, ^266^
CCBs	IIb	B	^255^, ^265^
CMVD without myocardial hypertrophy			
ACEIs/ARBs	I	A	^143^, ^267^
Beta‐blockers	I	A	^268^, ^269^, ^270^, ^271^
Allopurinol	IIb	B	^272^

Abbreviations: ACEIs, angiotensin‐converting enzyme inhibitors; ARBs, angiotensin receptor blockers; CMVD, coronary microvascular disease; CCB, calcium channel blocker.

## 7 GAPS OF KNOWLEDGE AND FUTURE PERSPECTIVES

There exists significant gap of knowledge in the field of CMVD. First, current knowledge of pathogenic mechanism of CMVD was based on findings in animal models and vascular cells of aortic atherosclerosis, which is in many ways different from CMVD. Development of animal models with CMVD and culture of microvascular endothelial cells from these models are essential for further understanding of the pathogenesis of CMVD. Second, epidemiological data of CMVD in different populations and ethnics, and in patients with less common etiologies, such as those with cardiomyopathy, are lacking. Thus, further epidemiological studies in these populations are required. Third, standard techniques for detecting CMVD are mostly invasive and focusing on only LAD and its supplied territories. Thus, development of noninvasive imaging techniques to evaluate the structure and function of entire left ventricular microvasculature is highly warranted. Finally, at present, all published randomized clinical trials for assessing therapeutic effects of drugs in patients with CMVD used surrogate endpoint, mostly CFR, and clinical trials using hard endpoint, such as cardiovascular mortality, should be the future direction.

## AUTHOR CONTRIBUTION

Wenqiang Chen, Mei Ni, He Huang, and Yundai Chen drafted the manuscript. Hongliang Cong, Xianghua Fu, Wei Gao, Yuejin Yang, Mengyue Yu, Xiantao Song, Meilin Liu, Zuyi Yuan, Bo Zhang, Zhaohui Wang, and Yan Wang participated in the writing and revision of the manuscript. Cheng Zhang and Yun Zhang designed the structure and contents of the consensus and corrected the manuscript. We confirmed that all authors have read and approved the final manuscript.

## CONFLICT OF INTEREST STATEMENT

All participants in the preparation of the manuscript declare that there is no conflict of interest relevant to this consensus.

## ETHICS STATEMENT

Not relevant.

## Data Availability

All data associated with this consensus are within the paper itself.

## References

[1] Likoff W , Segal BL , Kasparian H . Paradox of normal selective coronary arteriograms in patients considered to have unmistakable coronary heart disease. N Engl J Med. 1967;276(19):1063‐1066. 6025663 10.1056/NEJM196705112761904

[2] Task Force M , Montalescot G , Sechtem U , et al. ESC guidelines on the management of stable coronary artery disease: the Task Force on the management of stable coronary artery disease of the European Society of Cardiology. Eur Heart J. 2013;34(38):2949‐3003. 2013. 23996286 10.1093/eurheartj/eht296

[3] Yun Z , Yundai C , Xianghua F , et al. Chinese expert consensus on the diagnosis and treatment of coronary artery microvascular disease. Chin Circ J. 2017;32(5):421‐430.

[4] Ong P , Camici PG , Beltrame JF , et al. International standardization of diagnostic criteria for microvascular angina. Int J Cardiol. 2018;250:16‐20. 29031990 10.1016/j.ijcard.2017.08.068

[5] Knuuti J , Wijns W , Saraste A , et al. ESC Guidelines for the diagnosis and management of chronic coronary syndromes. Eur Heart J. 2019;41(3):407‐477. 10.1093/eurheartj/ehz425 31504439

[6] Tamis‐Holland JE , Jneid H , Reynolds HR , et al. Contemporary diagnosis and management of patients with myocardial infarction in the absence of obstructive coronary artery disease: a scientific statement from the. American Heart Association. Circulation. 2019;139(18):e891‐e908. 30913893 10.1161/CIR.0000000000000670

[7] Kunadian V , Chieffo A , Camici PG , et al. An EAPCI expert consensus document on ischaemia with non‐obstructive coronary arteries in collaboration with European Society of Cardiology Working Group on coronary pathophysiology & microcirculation endorsed by Coronary Vasomotor Disorders International Study Group. Eur Heart J. 2020;41(37):3504‐3520. 32626906 10.1093/eurheartj/ehaa503 PMC7577516

[8] Padro T , Manfrini O , Bugiardini R , et al. ESC. Cardiovasc Res. 2020;116(4):741‐755. 32034397 10.1093/cvr/cvaa003 PMC7825482

[9] Jacobs AK , Kushner FG , Ettinger SM , et al. ACCF/AHA clinical practice guideline methodology summit report: a report of the American College of Cardiology Foundation/American Heart Association Task Force on Practice Guidelines. Circulation. 2013;127(2):268‐310. 23230312 10.1161/CIR.0b013e31827e8e5f

[10] Gdowski MA , Murthy VL , Doering M , Monroy‐Gonzalez AG , Slart R , Brown DL . Association of isolated coronary microvascular dysfunction with mortality and major adverse cardiac events: a systematic review and meta‐analysis of aggregate data. J Am Heart Assoc. 2020;9(9):e014954. 32345133 10.1161/JAHA.119.014954 PMC7428565

[11] Taqueti VR , Hachamovitch R , Murthy VL , et al. Global coronary flow reserve is associated with adverse cardiovascular events independently of luminal angiographic severity and modifies the effect of early revascularization. Circulation. 2015;131(1):19‐27. 25400060 10.1161/CIRCULATIONAHA.114.011939 PMC4286486

[12] Pepine CJ , Anderson RD , Sharaf BL , et al. Coronary microvascular reactivity to adenosine predicts adverse outcome in women evaluated for suspected ischemia results from the National Heart, Lung and Blood Institute WISE (Women's Ischemia Syndrome Evaluation) study. J Am Coll Cardiol. 2010;55(25):2825‐2832. 20579539 10.1016/j.jacc.2010.01.054 PMC2898523

[13] Shimokawa H , Suda A , Takahashi J , et al. Clinical characteristics and prognosis of patients with microvascular angina: an international and prospective cohort study by the Coronary Vasomotor Disorders International Study (COVADIS) Group. Eur Heart J. 2021;42(44):4592‐4600. 34038937 10.1093/eurheartj/ehab282 PMC8633728

[14] Peng C , Nie S , Sun Y , et al. Non‐obstructive coronary artery disease in Chinese patients with angina diagnosed by coronary angiography: a retrospective study. Cardiol Discov. 2021;1:223‐227.

[15] Mileva N , Nagumo S , Mizukami T , et al. Prevalence of coronary microvascular disease and coronary vasospasm in patients with nonobstructive coronary artery disease: systematic review and meta‐analysis. J Am Heart Assoc. 2022;11(7):e023207. 35301851 10.1161/JAHA.121.023207 PMC9075440

[16] Canu M , Khouri C , Marliere S , et al. Prognostic significance of severe coronary microvascular dysfunction post‐PCI in patients with STEMI: a systematic review and meta‐analysis. PLoS One. 2022;17(5):e0268330. 35576227 10.1371/journal.pone.0268330 PMC9109915

[17] Pasupathy S , Air T , Dreyer RP , Tavella R , Beltrame JF . Systematic review of patients presenting with suspected myocardial infarction and nonobstructive coronary arteries. Circulation. 2015;131(10):861‐870. 25587100 10.1161/CIRCULATIONAHA.114.011201

[18] Reynolds HR , Srichai MB , Iqbal SN , et al. Mechanisms of myocardial infarction in women without angiographically obstructive coronary artery disease. Circulation. 2011;124(13):1414‐1425. 21900087 10.1161/CIRCULATIONAHA.111.026542 PMC3619391

[19] Pirozzolo G , Seitz A , Athanasiadis A , Bekeredjian R , Sechtem U , Ong P . Microvascular spasm in non‐ST‐segment elevation myocardial infarction without culprit lesion (MINOCA). Clin Res Cardiol. 2020;109(2):246‐254. 31236694 10.1007/s00392-019-01507-w

[20] Cenko E , van der Schaar M , Yoon J , et al. Sex‐Specific treatment effects after primary percutaneous intervention: a study on coronary blood flow and delay to hospital presentation. J Am Heart Assoc. 2019;8(4):e011190. 30764687 10.1161/JAHA.118.011190 PMC6405653

[21] Nishi T , Murai T , Ciccarelli G , et al. Prognostic value of coronary microvascular function measured immediately after percutaneous coronary intervention in stable coronary artery disease: an international multicenter study. Circ Cardiovasc Interv. 2019;12(9):e007889. 31525096 10.1161/CIRCINTERVENTIONS.119.007889

[22] Seraphim A , Dowsing B , Rathod KS , et al. Quantitative myocardial perfusion predicts outcomes in patients with prior surgical revascularization. J Am Coll Cardiol. 2022;79(12):1141‐1151. 35331408 10.1016/j.jacc.2021.12.037 PMC9034686

[23] Spyrou N , Khan MA , Rosen SD , et al. Persistent but reversible coronary microvascular dysfunction after bypass grafting. Am J Physiol Heart Circ Physiol. 2000;279(6):H2634‐H2640. 11087215 10.1152/ajpheart.2000.279.6.H2634

[24] Pelliccia F , Cecchi F , Olivotto I , Camici PG . Microvascular dysfunction in hypertrophic cardiomyopathy. J Clin Med. 2022;11(21):6560. 36362787 10.3390/jcm11216560 PMC9658510

[25] Wada T , Shiono Y , Honda K , et al. Serial changes of coronary flow reserve over one year after transcatheter aortic valve implantation in patients with severe aortic stenosis. Int J Cardiol Heart Vasc. 2022;42:101090. 35873862 10.1016/j.ijcha.2022.101090 PMC9304717

[26] Dorbala S , Vangala D , Bruyere J , et al. Coronary microvascular dysfunction is related to abnormalities in myocardial structure and function in cardiac amyloidosis. JACC Heart Fail. 2014;2(4):358‐367. 25023822 10.1016/j.jchf.2014.03.009 PMC4127145

[27] Shah SJ , Lam CSP , Svedlund S , et al. Prevalence and correlates of coronary microvascular dysfunction in heart failure with preserved ejection fraction: pROMIS‐HFpEF. Eur Heart J. 2018;39(37):3439‐3450. 30165580 10.1093/eurheartj/ehy531 PMC6927847

[28] Montone RA , Galiuto L , Meucci MC , et al. Coronary slow flow is associated with a worse clinical outcome in patients with Takotsubo syndrome. Heart. 2020;106(12):923‐930. 31924712 10.1136/heartjnl-2019-315909

[29] Murthy VL , Naya M , Foster CR , et al. Association between coronary vascular dysfunction and cardiac mortality in patients with and without diabetes mellitus. Circulation. 2012;126(15):1858‐1868. 22919001 10.1161/CIRCULATIONAHA.112.120402 PMC3495105

[30] Rigo F , Gherardi S , Galderisi M , et al. The prognostic impact of coronary flow‐reserve assessed by Doppler echocardiography in non‐ischaemic dilated cardiomyopathy. Eur Heart J. 2006;27(11):1319‐1323. 16464914 10.1093/eurheartj/ehi795

[31] Galea N , Rosato E , Gigante A , et al. Early myocardial damage and microvascular dysfunction in asymptomatic patients with systemic sclerosis: a cardiovascular magnetic resonance study with cold pressor test. PLoS One. 2020;15(12):e0244282. 33351821 10.1371/journal.pone.0244282 PMC7755221

[32] Liao KP , Huang J , He Z , et al. Coronary microvascular dysfunction in rheumatoid arthritis compared to diabetes mellitus and association with all‐cause mortality. Arthritis Care Res (Hoboken). 2021;73(2):159‐165. 31705724 10.1002/acr.24108 PMC7210065

[33] Tomanek, RJ Coronary vasculature || Structure–function of the coronary hierarchy. 2013;10.1007/978‐1‐4614‐4887‐7(Chapter 4):59‐81.

[34] Deussen A , Ohanyan V , Jannasch A , Yin L , Chilian W . Mechanisms of metabolic coronary flow regulation. J Mol Cell Cardiol. 2012;52(4):794‐801. 22004900 10.1016/j.yjmcc.2011.10.001

[35] Kassab GS , Lin DH , Fung YC . Morphometry of pig coronary venous system. Am J Physiol. 1994;267(6):H2100‐H2113. Pt 2. 7810711 10.1152/ajpheart.1994.267.6.H2100

[36] Jones CJ , Kuo L , Davis MJ , Chilian WM . Regulation of coronary blood flow: coordination of heterogeneous control mechanisms in vascular microdomains. Cardiovasc Res. 1995;29(5):585‐596. 7606744

[37] Chilian WM . Coronary microcirculation in health and disease. Summary of an NHLBI workshop. Circulation. 1997;95(2):522‐528. 9008472 10.1161/01.cir.95.2.522 PMC4037233

[38] Hoffman JI . A critical view of coronary reserve. Circulation. 1987;75(1):I6‐I11. Pt 2. 2947752

[39] Inoue K , Hamada M , Ohtsuka T , et al. Myocardial microvascular abnormalities observed by intravenous myocardial contrast echocardiography in patients with hypertrophic cardiomyopathy. Am J Cardiol. 2004;94(1):55‐58. 15219509 10.1016/j.amjcard.2004.03.030

[40] Yannoutsos A , Levy BI , Safar ME , Slama G , Blacher J . Pathophysiology of hypertension: interactions between macro and microvascular alterations through endothelial dysfunction. J Hypertens. 2014;32(2):216‐224. 24270179 10.1097/HJH.0000000000000021

[41] Cardiovascular Disease Branch of China International Exchange and Promotive Association for Medical and Healthcare. Chinese experts agree on blood pressure management in patients with hypertension complicated with coronary heart disease (press in Chinese). Zhonghua Yi Xue Za Zhi. 2022;102(10):717‐728.

[42] Camici PG , Tschope C , Di Carli MF , Rimoldi O , Van Linthout S . Coronary microvascular dysfunction in hypertrophy and heart failure. Cardiovasc Res. 2020;116(4):806‐816. 31999329 10.1093/cvr/cvaa023

[43] Porto I , Belloni F , Niccoli G , et al. Filter no‐reflow during percutaneous coronary intervention of saphenous vein grafts: incidence, predictors and effect of the type of protection device. EuroIntervention. 2011;7(8):955‐961. 22157481 10.4244/EIJV7I8A151

[44] Reinstadler SJ , Stiermaier T , Fuernau G , et al. The challenges and impact of microvascular injury in ST‐elevation myocardial infarction. Expert Rev Cardiovasc Ther. 2016;14(4):431‐443. 26794717 10.1586/14779072.2016.1135055

[45] Robbers LF , Eerenberg ES , Teunissen PF , et al. Magnetic resonance imaging‐defined areas of microvascular obstruction after acute myocardial infarction represent microvascular destruction and haemorrhage. Eur Heart J. 2013;34(30):2346‐2353. 23594591 10.1093/eurheartj/eht100

[46] Jaffe R , Dick A , Strauss BH . Prevention and treatment of microvascular obstruction‐related myocardial injury and coronary no‐reflow following percutaneous coronary intervention: a systematic approach. JACC Cardiovasc Interv. 2010;3(7):695‐704. 20650430 10.1016/j.jcin.2010.05.004

[47] Godo S , Suda A , Takahashi J , Yasuda S , Shimokawa H . Coronary microvascular dysfunction. Arterioscler Thromb Vasc Biol. 2021;41(5):1625‐1637. 33761763 10.1161/ATVBAHA.121.316025

[48] Sechtem U , Brown D , Godo S , Lanza GA , Shimokawa H , Sidik N . Coronary microvascular dysfunction in stable ischaemic heart disease (non‐obstructive coronary artery disease and obstructive coronary artery disease). Cardiovasc Res. 2020;116(4):771‐786. 31958128 10.1093/cvr/cvaa005

[49] Taqueti VR , Di Carli MF . Coronary microvascular disease pathogenic mechanisms and therapeutic options: jACC state‐of‐the‐art review. J Am Coll Cardiol. 2018;72(21):2625‐2641. 30466521 10.1016/j.jacc.2018.09.042 PMC6296779

[50] Ong P , Athanasiadis A , Borgulya G , Mahrholdt H , Kaski JC , Sechtem U . High prevalence of a pathological response to acetylcholine testing in patients with stable angina pectoris and unobstructed coronary arteries. The ACOVA Study (Abnormal COronary VAsomotion in patients with stable angina and unobstructed coronary arteries). J Am Coll Cardiol. 2012;59(7):655‐662. 22322081 10.1016/j.jacc.2011.11.015

[51] Shimokawa H . 2014. Williams Harvey Lecture: importance of coronary vasomotion abnormalities from bench to bedside. Eur Heart J. 2014;35(45):3180‐3193. 25354517 10.1093/eurheartj/ehu427

[52] Odaka Y , Takahashi J , Tsuburaya R , et al. Plasma concentration of serotonin is a novel biomarker for coronary microvascular dysfunction in patients with suspected angina and unobstructive coronary arteries. Eur Heart J. 2017;38(7):489‐496. 27694191 10.1093/eurheartj/ehw448

[53] Corban MT , Lerman LO , Lerman A . Endothelin‐1 in coronary microvascular dysfunction: a potential new therapeutic target once again. Eur Heart J. 2020;41(34):3252‐3254. 32031581 10.1093/eurheartj/ehz954

[54] Vancheri F , Longo G , Vancheri S , Henein M . Coronary microvascular dysfunction. J Clin Med. 2020;9(9):2880. 32899944 10.3390/jcm9092880 PMC7563453

[55] Mejia‐Renteria H , Travieso A , Matias‐Guiu JA , et al. Coronary microvascular dysfunction is associated with impaired cognitive function: the Cerebral‐Coronary Connection study (C3 study). Eur Heart J. 2023;44(2):113‐125. 36337036 10.1093/eurheartj/ehac521 PMC9825810

[56] Webb CM , Collins P , Di Mario C . Normal coronary physiology assessed by intracoronary Doppler ultrasound. Herz. 2005;30(1):8‐16. 15754151 10.1007/s00059-005-2647-z

[57] McGuinness ME , Talbert RL . Pharmacologic stress testing: experience with dipyridamole, adenosine, and dobutamine. Am J Hosp Pharm. 1994;51(3):328‐346. 8160685

[58] Chareonthaitawee P , Kaufmann PA , Rimoldi O , Camici PG . Heterogeneity of resting and hyperemic myocardial blood flow in healthy humans. Cardiovasc Res. 2001;50(1):151‐161. 11282088 10.1016/s0008-6363(01)00202-4

[59] JCS Joint Working Group . Guidelines for diagnosis and treatment of patients with vasospastic angina (coronary spastic angina) (JCS 2008): digest version. Circ J. 2010;74(8):1745‐1762. 20671373 10.1253/circj.cj-10-74-0802

[60] Del Buono MG , Montone RA , Camilli M , et al. Coronary microvascular dysfunction across the spectrum of cardiovascular diseases: jACC state‐of‐the‐art review. J Am Coll Cardiol. 2021;78(13):1352‐1371. 34556322 10.1016/j.jacc.2021.07.042 PMC8528638

[61] Chinese Society of Cardiology , Chinese Medical Association, & Editorial Board of Chinese Journal of Cardiology. Chinese expert consensus on microvascular protection strategy during emergency PCI therapy in patients with ST‐elevation myocardial infarction. Zhonghua Xin Xue Guan Bing Za Zhi. 2022;50(3):221‐230. 35340140 10.3760/cma.j.cn112148-20211112-00987

[62] Cardiovascular Disease Branch of . Chinese Geriatric Society. Chinese multidisciplinary expert consensus on the clinical diagnosis and treatment of microvascular diseases (press in Chinese). Zhong Guo Xun Huan Za Zhi. 2020;35(12):1149‐1165.

[63] Lethen H , PT H , , Kersting S , Lambertz H . Validation of noninvasive assessment of coronary flow velocity reserve in the right coronary artery. A comparison of transthoracic echocardiographic results with intracoronary Doppler flow wire measurements. Eur Heart J. 2003;24(17):1567‐1575. 12927192 10.1016/s0195-668x(03)00284-7

[64] Vegsundvag J , Holte E , Wiseth R , Hegbom K , Hole T . Coronary flow velocity reserve in the three main coronary arteries assessed with transthoracic Doppler: a comparative study with quantitative coronary angiography. J Am Soc Echocardiogr. 2011;24(7):758‐767. 21524564 10.1016/j.echo.2011.03.010

[65] Ong P , Safdar B , Seitz A , Hubert A , Beltrame JF , Prescott E . Diagnosis of coronary microvascular dysfunction in the clinic. Cardiovasc Res. 2020;116(4):841‐855. 31904824 10.1093/cvr/cvz339

[66] Wei K , Jayaweera AR , Firoozan S , Linka A , Skyba DM , Kaul S . Quantification of myocardial blood flow with ultrasound‐induced destruction of microbubbles administered as a constant venous infusion. Circulation. 1998;97(5):473‐483. 9490243 10.1161/01.cir.97.5.473

[67] Vogel R , Indermuhle A , Reinhardt J , et al. The quantification of absolute myocardial perfusion in humans by contrast echocardiography: algorithm and validation. J Am Coll Cardiol. 2005;45(5):754‐762. 15734622 10.1016/j.jacc.2004.11.044

[68] Feher A , Chen SY , Bagi Z , Arora V . Prevention and treatment of no‐reflow phenomenon by targeting the coronary microcirculation. Rev Cardiovasc Med. 2014;15(1):38‐51. 24762465 10.3909/ricm0699

[69] Liu C , Sinusas AJ . Is assessment of absolute myocardial perfusion with SPECT ready for prime time? J Nucl Med. 2014;55(10):1573‐1575. 25236351 10.2967/jnumed.114.144550

[70] Zavadovsky KV , Mochula AV , Maltseva AN , et al. The current status of CZT SPECT myocardial blood flow and reserve assessment: tips and tricks. J Nucl Cardiol. 2022;29(6):3137‐3151. 33939162 10.1007/s12350-021-02620-y

[71] Bergmann SR , Fox KA , Rand AL , et al. Quantification of regional myocardial blood flow in vivo with H215O. Circulation. 1984;70(4):724‐733. 6332687 10.1161/01.cir.70.4.724

[72] Bol A , Melin JA , Vanoverschelde JL , et al. Direct comparison of [13N]ammonia and [15O]water estimates of perfusion with quantification of regional myocardial blood flow by microspheres. Circulation. 1993;87(2):512‐525. 8425298 10.1161/01.cir.87.2.512

[73] Fonti R , Conson M , Del Vecchio S . PET/CT in radiation oncology. Semin Oncol. 2019;46(3):202‐209. 31378377 10.1053/j.seminoncol.2019.07.001

[74] Rischpler C , Nekolla SG , Heusch G , et al. Cardiac PET/MRI‐an update. Eur J Hybrid Imaging. 2019;3(1):2. 34191143 10.1186/s41824-018-0050-2 PMC8212244

[75] Schepis T , Gaemperli O , Treyer V , et al. Absolute quantification of myocardial blood flow with 13N‐ammonia and 3‐dimensional PET. J Nucl Med. 2007;48(11):1783‐1789. 17942816 10.2967/jnumed.107.044099

[76] Nesterov SV , Deshayes E , Sciagra R , et al. Quantification of myocardial blood flow in absolute terms using (82)Rb PET imaging: the RUBY‐10 Study. JACC Cardiovasc Imaging. 2014;7(11):1119‐1127. 25306543 10.1016/j.jcmg.2014.08.003 PMC4260449

[77] Di Bella EV , Parker DL , Sinusas AJ . On the dark rim artifact in dynamic contrast‐enhanced MRI myocardial perfusion studies. Magn Reson Med. 2005;54(5):1295‐1299. 16200553 10.1002/mrm.20666 PMC2377407

[78] Brown LAE , Onciul SC , Broadbent DA , et al. Fully automated, inline quantification of myocardial blood flow with cardiovascular magnetic resonance: repeatability of measurements in healthy subjects. J Cardiovasc Magn Reson. 2018;20(1):48. 29983119 10.1186/s12968-018-0462-y PMC6036695

[79] Kotecha T , Martinez‐Naharro A , Boldrini M , et al. Automated Pixel‐Wise quantitative myocardial perfusion mapping by CMR to detect obstructive coronary artery disease and coronary microvascular dysfunction: validation against invasive coronary physiology. JACC Cardiovasc Imaging. 2019;12(10):1958‐1969. 30772231 10.1016/j.jcmg.2018.12.022 PMC8414332

[80] Engblom H , Xue H , Akil S , et al. Fully quantitative cardiovascular magnetic resonance myocardial perfusion ready for clinical use: a comparison between cardiovascular magnetic resonance imaging and positron emission tomography. J Cardiovasc Magn Reson. 2017;19(1):78. 29047385 10.1186/s12968-017-0388-9 PMC5648469

[81] Rossi A , Merkus D , Klotz E , Mollet N , de Feyter PJ , Krestin GP . Stress myocardial perfusion: imaging with multidetector CT. Radiology. 2014;270(1):25‐46. 24354374 10.1148/radiol.13112739

[82] Danad I , Szymonifka J , Schulman‐Marcus J , Min JK . Static and dynamic assessment of myocardial perfusion by computed tomography. Eur Heart J Cardiovasc Imaging. 2016;17(8):836‐844. 27013250 10.1093/ehjci/jew044 PMC4955293

[83] Nakamura S , Kitagawa K , Goto Y , et al. Incremental prognostic value of myocardial blood flow quantified with stress dynamic computed tomography perfusion imaging. JACC Cardiovasc Imaging. 2019;12(7):1379‐1387. Pt 2. 30031698 10.1016/j.jcmg.2018.05.021

[84] Rochitte CE , George RT , Chen MY , et al. Computed tomography angiography and perfusion to assess coronary artery stenosis causing perfusion defects by single photon emission computed tomography: the CORE320 study. Eur Heart J. 2014;35(17):1120‐1130. 24255127 10.1093/eurheartj/eht488 PMC6693293

[85] Andreini D , Mushtaq S , Pontone G , et al. CT Perfusion versus coronary CT angiography in patients with suspected In‐Stent restenosis or CAD progression. JACC Cardiovasc Imaging. 2020;13(3):732‐742. 31422127 10.1016/j.jcmg.2019.05.031

[86] TIMI Study Group.The thrombolysis in myocardial infarction (TIMI) trial. Phase I findings. N Engl J Med. 1985;312(14):932‐936. 4038784 10.1056/NEJM198504043121437

[87] Gibson CM , Cannon CP , Daley WL , et al. TIMI frame count: a quantitative method of assessing coronary artery flow. Circulation. 1996;93(5):879‐888. 8598078 10.1161/01.cir.93.5.879

[88] Molloi S , Ersahin A , Tang J , Hicks J , Leung CY . Quantification of volumetric coronary blood flow with dual‐energy digital subtraction angiography. Circulation. 1996;93(10):1919‐1927. 8635272 10.1161/01.cir.93.10.1919

[89] van ’t Hof AW , Liem A , Suryapranata H , Hoorntje JC , de Boer MJ , Zijlstra F . Angiographic assessment of myocardial reperfusion in patients treated with primary angioplasty for acute myocardial infarction: myocardial blush grade. Zwolle Myocardial Infarction Study Group. Circulation. 1998;97(23):2302‐2306. 9639373 10.1161/01.cir.97.23.2302

[90] Ding S , Pu J , Qiao ZQ , et al. TIMI myocardial perfusion frame count: a new method to assess myocardial perfusion and its predictive value for short‐term prognosis. Catheter Cardiovasc Interv. 2010;75(5):722‐732. 19960517 10.1002/ccd.22298

[91] Ge H , Ding S , An D , et al. Frame counting improves the assessment of post‐reperfusion microvascular patency by TIMI myocardial perfusion grade: evidence from cardiac magnetic resonance imaging. Int J Cardiol. 2016;203:360‐366. 26539957 10.1016/j.ijcard.2015.10.194

[92] Montone RA , Meucci MC , De Vita A , Lanza GA , Niccoli G . Coronary provocative tests in the catheterization laboratory: pathophysiological bases, methodological considerations and clinical implications. Atherosclerosis. 2021;318:14‐21. 33360263 10.1016/j.atherosclerosis.2020.12.008

[93] Crea F , Montone RA , Rinaldi R . Pathophysiology of coronary microvascular dysfunction. Circ J. 2022;86(9):1319‐1328. 34759123 10.1253/circj.CJ-21-0848

[94] Montone RA , Rinaldi R , Del Buono MG , et al. Safety and prognostic relevance of acetylcholine testing in patients with stable myocardial ischaemia or myocardial infarction and non‐obstructive coronary arteries. EuroIntervention. 2022;18(8):e666‐e676. 35377315 10.4244/EIJ-D-21-00971 PMC10241282

[95] Pijls NH , De Bruyne B , Smith L , et al. Coronary thermodilution to assess flow reserve: validation in humans. Circulation. 2002;105(21):2482‐2486. 12034653 10.1161/01.cir.0000017199.09457.3d

[96] Fearon WF , Balsam LB , Farouque HM , et al. Novel index for invasively assessing the coronary microcirculation. Circulation. 2003;107(25):3129‐3132. 12821539 10.1161/01.CIR.0000080700.98607.D1

[97] Mangiacapra F , Peace AJ , Di Serafino L , et al. Intracoronary enalaprilat to reduce microvascular damage during percutaneous coronary intervention (ProMicro) study. J Am Coll Cardiol. 2013;61(6):615‐621. 23290547 10.1016/j.jacc.2012.11.025

[98] Ng MK , Yeung AC , Fearon WF . Invasive assessment of the coronary microcirculation: superior reproducibility and less hemodynamic dependence of index of microcirculatory resistance compared with coronary flow reserve. Circulation. 2006;113(17):2054‐2061. 16636168 10.1161/CIRCULATIONAHA.105.603522

[99] Adjedj J , Picard F , Collet C , et al. Intracoronary saline‐induced hyperemia during coronary thermodilution measurements of absolute coronary blood flow: an animal mechanistic study. J Am Heart Assoc. 2020;9(15):e015793. 32689859 10.1161/JAHA.120.015793 PMC7792254

[100] Jansen TPJ , Konst RE , Elias‐Smale SE , et al. Assessing microvascular dysfunction in angina with unobstructed coronary arteries: jACC review topic of the week. J Am Coll Cardiol. 2021;78(14):1471‐1479. 34593129 10.1016/j.jacc.2021.08.028

[101] Everaars H , de Waard GA , Schumacher SP , et al. Continuous thermodilution to assess absolute flow and microvascular resistance: validation in humans using [15O]H2O positron emission tomography. Eur Heart J. 2019;40(28):2350‐2359. 31327012 10.1093/eurheartj/ehz245

[102] De Bruyne B , Pijls NHJ , Gallinoro E , et al. Microvascular resistance reserve for assessment of coronary microvascular function: jACC Technology Corner. J Am Coll Cardiol. 2021;78(15):1541‐1549. 34620412 10.1016/j.jacc.2021.08.017

[103] Barbato E , Aarnoudse W , Aengevaeren WR , et al. Validation of coronary flow reserve measurements by thermodilution in clinical practice. Eur Heart J. 2004;25(3):219‐223. 14972422 10.1016/j.ehj.2003.11.009

[104] Jansen TPJ , de Vos A , Paradies V , et al. Continuous versus bolus thermodilution‐derived coronary flow reserve and microvascular resistance reserve and their association with angina and quality of life in patients with angina and nonobstructive coronaries: a head‐to‐head comparison. J Am Heart Assoc. 2023;12(16):e030480. 37577948 10.1161/JAHA.123.030480 PMC10492956

[105] de Vos A , Jansen TPJ , van ’t Veer M , et al. Microvascular resistance reserve to assess microvascular dysfunction in ANOCA Patients. JACC Cardiovasc Interv. 2023;16(4):470‐481. 36858668 10.1016/j.jcin.2022.12.012

[106] Boerhout CKM , Lee JM , de Waard GA , et al. Microvascular resistance reserve: diagnostic and prognostic performance in the ILIAS registry. Eur Heart J. 2023;44(30):2862‐2869. 37350567 10.1093/eurheartj/ehad378 PMC10406337

[107] Paolisso P , Gallinoro E , Vanderheyden M , et al. Absolute coronary flow and microvascular resistance reserve in patients with severe aortic stenosis. Heart. 2022;109(1):47‐54. 35977812 10.1136/heartjnl-2022-321348

[108] Mei Z , Yun Z . The Study of the measurement of coronary flow reserve by intracoronary injection of papaverine (press in Chinese). Zhong Guo Chao Sheng Yi Xue Za Zhi. 1998;14(6):17‐19.

[109] Kaski JC , Crea F , Gersh BJ , Camici PG . Reappraisal of ischemic heart disease. Circulation. 2018;138(14):1463‐1480. 30354347 10.1161/CIRCULATIONAHA.118.031373

[110] Camici PG , Crea F . Coronary microvascular dysfunction. N Engl J Med. 2007;356(8):830‐840. 17314342 10.1056/NEJMra061889

[111] Xaplanteris P , Fournier S , Keulards DCJ , et al. Catheter‐based measurements of absolute coronary blood flow and microvascular resistance: feasibility, safety, and reproducibility in humans. Circ Cardiovasc Interv. 2018;11(3):e006194. 29870386 10.1161/CIRCINTERVENTIONS.117.006194

[112] Xie F , Dodla S , O'Leary E , Porter TR . Detection of subendocardial ischemia in the left anterior descending coronary artery territory with real‐time myocardial contrast echocardiography during dobutamine stress echocardiography. JACC Cardiovasc Imaging. 2008;1(3):271‐278. 19356438 10.1016/j.jcmg.2008.02.004

[113] Taqui S , Ferencik M , Davidson BP , et al. Coronary microvascular dysfunction by myocardial contrast echocardiography in nonelderly patients referred for computed tomographic coronary angiography. J Am Soc Echocardiogr. 2019;32(7):817‐825. 31103385 10.1016/j.echo.2019.03.001 PMC6527356

[114] Tona F , Montisci R , Iop L , Civieri G . Role of coronary microvascular dysfunction in heart failure with preserved ejection fraction. Rev Cardiovasc Med. 2021;22(1):97‐104. 33792251 10.31083/j.rcm.2021.01.277

[115] Brainin P , Frestad D , Prescott E . The prognostic value of coronary endothelial and microvascular dysfunction in subjects with normal or non‐obstructive coronary artery disease: a systematic review and meta‐analysis. Int J Cardiol. 2018;254:1‐9. 29407076 10.1016/j.ijcard.2017.10.052

[116] Shah NR , Cheezum MK , Veeranna V , et al. Ranolazine in symptomatic diabetic patients without obstructive coronary artery disease: impact on microvascular and diastolic function. J Am Heart Assoc. 2017;6(5):e005027. 28473401 10.1161/JAHA.116.005027 PMC5524071

[117] Klein R , Ocneanu A , Renaud JM , Ziadi MC , Beanlands RSB , deKemp RA . Consistent tracer administration profile improves test‐retest repeatability of myocardial blood flow quantification with (82)Rb dynamic PET imaging. J Nucl Cardiol. 2018;25(3):929‐941. 27804067 10.1007/s12350-016-0698-6 PMC5966478

[118] Manka R , Wissmann L , Gebker R , et al. Multicenter evaluation of dynamic three‐dimensional magnetic resonance myocardial perfusion imaging for the detection of coronary artery disease defined by fractional flow reserve. Circ Cardiovasc Imaging. 2015;8(5):e003061. 25901043 10.1161/CIRCIMAGING.114.003061

[119] Li M , Zhou T , Yang LF , Peng ZH , Ding J , Sun G . Diagnostic accuracy of myocardial magnetic resonance perfusion to diagnose ischemic stenosis with fractional flow reserve as reference: systematic review and meta‐analysis. JACC Cardiovasc Imaging. 2014;7(11):1098‐1105. 25306540 10.1016/j.jcmg.2014.07.011

[120] Del Buono MG , Montone RA , Iannaccone G , et al. Diagnostic work‐up and therapeutic implications in MINOCA: need for a personalized approach. Future Cardiol. 2021;17(1):149‐154. 32628045 10.2217/fca-2020-0052

[121] Niccoli G , Montone RA , Ibanez B , et al. Optimized treatment of ST‐Elevation myocardial infarction. Circ Res. 2019;125(2):245‐258. 31268854 10.1161/CIRCRESAHA.119.315344

[122] Bulluck H , Foin N , Cabrera‐Fuentes HA , et al. Index of microvascular resistance and microvascular obstruction in patients with acute myocardial infarction. JACC Cardiovasc Interv. 2016;9(20):2172‐2174. 10.1016/j.jcin.2016.08.018 27765315

[123] Hess CN , Kaltenbach LA , Doll JA , Cohen DJ , Peterson ED , Wang TY . Race and sex differences in post‐myocardial infarction angina frequency and risk of 1‐year unplanned rehospitalization. Circulation. 2017;135(6):532‐543. 28153990 10.1161/CIRCULATIONAHA.116.024406

[124] De Maria GL , Cuculi F , Patel N , et al. How does coronary stent implantation impact on the status of the microcirculation during primary percutaneous coronary intervention in patients with ST‐elevation myocardial infarction? Eur Heart J. 2015;36(45):3165‐3177. 26254178 10.1093/eurheartj/ehv353 PMC4664836

[125] Cuisset T , Hamilos M , Melikian N , et al. Direct stenting for stable angina pectoris is associated with reduced periprocedural microcirculatory injury compared with stenting after pre‐dilation. J Am Coll Cardiol. 2008;51(11):1060‐1065. 18342222 10.1016/j.jacc.2007.11.059

[126] Mangiacapra F , Del Buono MG , Abbate A , et al. Role of endothelial dysfunction in determining angina after percutaneous coronary intervention: learning from pathophysiology to optimize treatment. Prog Cardiovasc Dis. 2020;63(3):233‐242. 32061633 10.1016/j.pcad.2020.02.009

[127] Serruys PW , di Mario C , Piek J , et al. Prognostic value of intracoronary flow velocity and diameter stenosis in assessing the short‐ and long‐term outcomes of coronary balloon angioplasty: the DEBATE Study (Doppler Endpoints Balloon Angioplasty Trial Europe). Circulation. 1997;96(10):3369‐3377. 9396429 10.1161/01.cir.96.10.3369

[128] Gahl B , Gober V , Odutayo A , et al. Prognostic value of early postoperative troponin T in patients undergoing coronary artery bypass grafting. J Am Heart Assoc. 2018;7(5):e007743. 29487111 10.1161/JAHA.117.007743 PMC5866325

[129] Nemes A , Balazs E , Csanady M , Forster T . Long‐term prognostic role of coronary flow velocity reserve in patients with aortic valve stenosis—insights from the SZEGED Study. Clin Physiol Funct Imaging. 2009;29(6):447‐452. 19712079 10.1111/j.1475-097X.2009.00893.x

[130] Beyerbacht HP , Lamb HJ , van Der Laarse A , et al. Aortic valve replacement in patients with aortic valve stenosis improves myocardial metabolism and diastolic function. Radiology. 2001;219(3):637‐643. 11376247 10.1148/radiology.219.3.r01jn25637

[131] Medina de Chazal H , Del Buono MG , Keyser‐Marcus L , et al. Stress cardiomyopathy diagnosis and treatment: jACC state‐of‐the‐art review. J Am Coll Cardiol. 2018;72(16):1955‐1971. 30309474 10.1016/j.jacc.2018.07.072 PMC7058348

[132] Galiuto L , De Caterina AR , Porfidia A , et al. Reversible coronary microvascular dysfunction: a common pathogenetic mechanism in Apical Ballooning or Tako‐Tsubo Syndrome. Eur Heart J. 2010;31(11):1319‐1327. 20215125 10.1093/eurheartj/ehq039

[133] Neglia D , Parodi O , Gallopin M , et al. Myocardial blood flow response to pacing tachycardia and to dipyridamole infusion in patients with dilated cardiomyopathy without overt heart failure. A quantitative assessment by positron emission tomography. Circulation. 1995;92(4):796‐804. 7641359 10.1161/01.cir.92.4.796

[134] Paulus WJ , Tschope C . A novel paradigm for heart failure with preserved ejection fraction: comorbidities drive myocardial dysfunction and remodeling through coronary microvascular endothelial inflammation. J Am Coll Cardiol. 2013;62(4):263‐271. 23684677 10.1016/j.jacc.2013.02.092

[135] Allan T , Dryer K , Fearon WF , Shah SJ , Blair JEA . Coronary microvascular dysfunction and clinical outcomes in patients with heart failure with preserved ejection fraction. J Card Fail. 2019;25(10):843‐845. 31487534 10.1016/j.cardfail.2019.08.010

[136] Dryer K , Gajjar M , Narang N , et al. Coronary microvascular dysfunction in patients with heart failure with preserved ejection fraction. Am J Physiol Heart Circ Physiol. 2018;314(5):H1033‐H1042. 29424571 10.1152/ajpheart.00680.2017 PMC6008137

[137] Yang JH , Obokata M , Reddy YNV , Redfield MM , Lerman A , Borlaug BA . Endothelium‐dependent and independent coronary microvascular dysfunction in patients with heart failure with preserved ejection fraction. Eur J Heart Fail. 2020;22(3):432‐441. 31840366 10.1002/ejhf.1671

[138] Nelson MD , Wei J , Bairey Merz CN . Coronary microvascular dysfunction and heart failure with preserved ejection fraction as female‐pattern cardiovascular disease: the chicken or the egg? Eur Heart J. 2018;39(10):850‐852. 29346550 10.1093/eurheartj/ehx818 PMC5939623

[139] Kristensen SL , Mogensen UM , Jhund PS , et al. Clinical and echocardiographic characteristics and cardiovascular outcomes according to diabetes status in patients with heart failure and preserved ejection fraction: a report from the I‐preserve trial (irbesartan in heart failure with preserved ejection fraction). Circulation. 2017;135(8):724‐735. 28052977 10.1161/CIRCULATIONAHA.116.024593

[140] Ford TJ , Stanley B , Good R , et al. Stratified medical therapy using invasive coronary function testing in angina: the CorMicA trial. J Am Coll Cardiol. 2018;72(23):2841‐2855. Pt A. 30266608 10.1016/j.jacc.2018.09.006

[141] Bove KB , Nilsson M , Pedersen LR , et al. Comprehensive treatment of microvascular angina in overweight women—a randomized controlled pilot trial. PLoS One. 2020;15(11):e0240722. 33151955 10.1371/journal.pone.0240722 PMC7644075

[142] Bairey Merz CN , Pepine CJ , Shimokawa H , Berry C . Treatment of coronary microvascular dysfunction. Cardiovasc Res. 2020;116(4):856‐870. 32087007 10.1093/cvr/cvaa006 PMC7061279

[143] Manfrini O , Morrell C , Das R , et al. Effects of angiotensin‐converting enzyme inhibitors and beta blockers on clinical outcomes in patients with and without coronary artery obstructions at angiography (from a register‐based cohort study on acute coronary syndromes). Am J Cardiol. 2014;113(10):1628‐1633. 24698468 10.1016/j.amjcard.2014.02.015

[144] Yong J , Tian J , Yang X , Xing H , He Y , Song X . Effects of oral drugs on coronary microvascular function in patients without significant stenosis of epicardial coronary arteries: a systematic review and meta‐analysis of coronary flow reserve. Front Cardiovasc Med. 2020;7:580419. 33195465 10.3389/fcvm.2020.580419 PMC7661556

[145] Ford TJ , Berry C . How to diagnose and manage angina without obstructive coronary artery disease: lessons from the British Heart Foundation CorMicA trial. Interv Cardiol. 2019;14(2):76‐82. 31178933 10.15420/icr.2019.04.R1 PMC6545998

[146] Ridker PM , MacFadyen J , Libby P , Glynn RJ . Relation of baseline high‐sensitivity C‐reactive protein level to cardiovascular outcomes with rosuvastatin in the justification for Use of statins in Prevention: an Intervention Trial Evaluating Rosuvastatin (JUPITER). Am J Cardiol. 2010;106(2):204‐209. 20599004 10.1016/j.amjcard.2010.03.018

[147] Zhang X , Li Q , Zhao J , et al. Effects of combination of statin and calcium channel blocker in patients with cardiac syndrome X. Coron Artery Dis. 2014;25(1):40‐44. 24256699 10.1097/MCA.0000000000000054

[148] Schremmer J , Busch L , Baasen S , et al. Chronic PCSK9 inhibitor therapy leads to sustained improvements in endothelial function, arterial stiffness, and microvascular function. Microvasc Res. 2023;148:104513. 36870561 10.1016/j.mvr.2023.104513

[149] Nicholls SJ , Puri R , Anderson T , et al. Effect of evolocumab on progression of coronary disease in statin‐treated patients: the GLAGOV randomized clinical trial. JAMA. 2016;316(22):2373‐2384. 27846344 10.1001/jama.2016.16951

[150] Ishihara M , Asakura M , Hibi K , et al. Evolocumab for prevention of microvascular dysfunction in patients undergoing percutaneous coronary intervention: the randomised, open‐label EVOCATION trial. EuroIntervention. 2022;18(8):e647‐e655. 35837711 10.4244/EIJ-D-22-00269 PMC10241273

[151] Carbone S , Dixon DL , Buckley LF , Abbate A . Glucose‐lowering therapies for cardiovascular risk reduction in type 2 diabetes mellitus: state‐of‐the‐art review. Mayo Clin Proc. 2018;93(11):1629‐1647. 30392544 10.1016/j.mayocp.2018.07.018 PMC6501786

[152] Jadhav S , Ferrell W , Greer IA , Petrie JR , Cobbe SM , Sattar N . Effects of metformin on microvascular function and exercise tolerance in women with angina and normal coronary arteries: a randomized, double‐blind, placebo‐controlled study. J Am Coll Cardiol. 2006;48(5):956‐963. 16949486 10.1016/j.jacc.2006.04.088

[153] Diabetes Prevention Program Research Group . Long‐term effects of lifestyle intervention or metformin on diabetes development and microvascular complications over 15‐year follow‐up: the diabetes prevention program outcomes study. Lancet Diabetes Endocrinol. 2015;3(11):866‐875. 26377054 10.1016/S2213-8587(15)00291-0 PMC4623946

[154] Ikonomidis I , Pavlidis G , Thymis J , et al. Effects of glucagon‐like peptide‐1 receptor agonists, sodium‐glucose cotransporter‐2 inhibitors, and their combination on endothelial glycocalyx, arterial function, and myocardial work index in patients with type 2 diabetes mellitus after 12‐month treatment. J Am Heart Assoc. 2020;9(9):e015716. 32326806 10.1161/JAHA.119.015716 PMC7428590

[155] Leccisotti L , Cinti F , Sorice GP , et al. Dapagliflozin improves myocardial flow reserve in patients with type 2 diabetes: the DAPAHEART Trial: a preliminary report. Cardiovasc Diabetol. 2022;21(1):173. 36057768 10.1186/s12933-022-01607-4 PMC9440459

[156] Braunwald E , Angiolillo D , Bates E , et al. The problem of persistent platelet activation in acute coronary syndromes and following percutaneous coronary intervention. Clin Cardiol. 2008;31(3):I17‐I20. Suppl 1. 18481817 10.1002/clc.20363 PMC6653003

[157] Baigent C , Blackwell L , Collins R , et al. Aspirin in the primary and secondary prevention of vascular disease: collaborative meta‐analysis of individual participant data from randomised trials. Lancet. 2009;373(9678):1849‐1860. 19482214 10.1016/S0140-6736(09)60503-1 PMC2715005

[158] Xu J , Lo S , Mussap CJ , et al. Impact of ticagrelor versus clopidogrel on coronary microvascular function after non‐ST‐segment‐elevation acute coronary syndrome. Circ Cardiovasc Interv. 2022;15(4):e011419. 35369712 10.1161/CIRCINTERVENTIONS.121.011419

[159] Rooks C , Faber T , Votaw J , et al. Effects of smoking on coronary microcirculatory function: a twin study. Atherosclerosis. 2011;215(2):500‐506. 21315354 10.1016/j.atherosclerosis.2011.01.012 PMC3082474

[160] Carbone S , Del Buono MG , Ozemek C , Lavie CJ . Obesity, risk of diabetes and role of physical activity, exercise training and cardiorespiratory fitness. Prog Cardiovasc Dis. 2019;62(4):327‐333. 31442513 10.1016/j.pcad.2019.08.004

[161] Olsen RH , Pedersen LR , Jürs A , Snoer M , Haugaard SB , Prescott E . A randomised trial comparing the effect of exercise training and weight loss on microvascular function in coronary artery disease. Int J Cardiol. 2015;185:229‐235. 25802037 10.1016/j.ijcard.2015.03.118

[162] Szot W , Zając J , Kubinyi A , Kostkiewicz M . The effects of cardiac rehabilitation on overall physical capacity and myocardial perfusion in women with microvascular angina. Kardiol Pol. 2016;74(5):431‐438. 26412475 10.5603/KP.a2015.0198

[163] Cattaneo M , Halasz G , Cattaneo MM , et al. The central nervous system and psychosocial factors in primary microvascular angina. Front Cardiovasc Med. 2022;9:896042. 35647077 10.3389/fcvm.2022.896042 PMC9136057

[164] Bugiardini R , Borghi A , Biagetti L , Puddu P . Comparison of verapamil versus propranolol therapy in syndrome X. Am J Cardiol. 1989;63(5):286‐290. 2643845 10.1016/0002-9149(89)90332-9

[165] Togni M , Vigorito F , Windecker S , et al. Does the beta‐blocker nebivolol increase coronary flow reserve? Cardiovasc Drugs Ther. 2007;21(2):99‐108. 17235472 10.1007/s10557-006-0494-7

[166] Massie BM . Mibefradil, a T‐type channel‐selective calcium antagonist: clinical trials in chronic stable angina pectoris. Am J Hypertens. 1998;11(4):95S‐102S. Pt 3. 9607373 10.1016/s0895-7061(98)00006-5

[167] Beltrame JF , Turner SP , Leslie SL , Solomon P , Freedman SB , Horowitz JD . The angiographic and clinical benefits of mibefradil in the coronary slow flow phenomenon. J Am Coll Cardiol. 2004;44(1):57‐62. 15234407 10.1016/j.jacc.2004.03.055

[168] Nishigaki K , Inoue Y , Yamanouchi Y , et al. Prognostic effects of calcium channel blockers in patients with vasospastic angina–a meta‐analysis. Circ J. 2010;74(9):1943‐1950. 20668353 10.1253/circj.cj-10-0292

[169] Cannon RO , Watson RM , Rosing DR , Epstein SE . Efficacy of calcium channel blocker therapy for angina pectoris resulting from small‐vessel coronary artery disease and abnormal vasodilator reserve. Am J Cardiol. 1985;56(4):242‐246. 4025160 10.1016/0002-9149(85)90842-2

[170] Ozçelik F , Altun A , Ozbay G . Antianginal and anti‐ischemic effects of nisoldipine and ramipril in patients with syndrome X. Clin Cardiol. 1999;22(5):361‐365. 10326170 10.1002/clc.4960220513 PMC6656278

[171] Hongo M , Takenaka H , Uchikawa S , Nakatsuka T , Watanabe N , Sekiguchi M . Coronary microvascular response to intracoronary administration of nicorandil. Am J Cardiol. 1995;75(4):246‐250. 7832132 10.1016/0002-9149(95)80029-r

[172] Yamabe H , Namura H , Yano T , et al. Effect of nicorandil on abnormal coronary flow reserve assessed by exercise 201Tl scintigraphy in patients with angina pectoris and nearly normal coronary arteriograms. Cardiovasc Drugs Ther. 1995;9(6):755‐761. 8850379 10.1007/BF00879868

[173] Mehta PK , Sharma S , Minissian M , et al. Ranolazine reduces angina in women with ischemic heart disease: results of an open‐label, multicenter trial. J Womens Health (Larchmt). 2019;28(5):573‐582. 30888919 10.1089/jwh.2018.7019 PMC6537111

[174] Ferrari R , Ford I , Fox K , et al. A randomized, double‐blind, placebo‐controlled trial to assess the efficAcy and safety of Trimetazidine in patients with angina pectoris having been treated by percutaneous coronary intervention (ATPCI study): rationale, design, and baseline characteristics. Am Heart J. 2019;210:98‐107. 30771737 10.1016/j.ahj.2018.12.015

[175] Pauly DF , Johnson BD , Anderson RD , et al. In women with symptoms of cardiac ischemia, nonobstructive coronary arteries, and microvascular dysfunction, angiotensin‐converting enzyme inhibition is associated with improved microvascular function: a double‐blind randomized study from the National Heart, Lung and Blood Institute Women's Ischemia Syndrome Evaluation (WISE). Am Heart J. 2011;162(4):678‐684. 21982660 10.1016/j.ahj.2011.07.011 PMC3191889

[176] Kawata T , Daimon M , Hasegawa R , et al. Effect on coronary flow velocity reserve in patients with type 2 diabetes mellitus: comparison between angiotensin‐converting enzyme inhibitor and angiotensin II type 1 receptor antagonist. Am Heart J. 2006;151(4):798.e9‐798.e15. 10.1016/j.ahj.2005.09.014 16569537

[177] Masuda D , Nohara R , Tamaki N , et al. Evaluation of coronary blood flow reserve by 13N‐NH3 positron emission computed tomography (PET) with dipyridamole in the treatment of hypertension with the ACE inhibitor (Cilazapril). Ann Nucl Med. 2000;14(5):353‐360. 11108164 10.1007/BF02988695

[178] Camici PG , Gloekler S , Levy BI , et al. Ivabradine in chronic stable angina: effects by and beyond heart rate reduction. Int J Cardiol. 2016;215:1‐6. 27104917 10.1016/j.ijcard.2016.04.001

[179] Morrow AJ , Ford TJ , Mangion K , et al. Rationale and design of the Medical Research Council's Precision Medicine with Zibotentan in Microvascular Angina (PRIZE) trial. Am Heart J. 2020;229:70‐80. 32942043 10.1016/j.ahj.2020.07.007 PMC7674581

[180] Cannon RO , Quyyumi AA , Mincemoyer R , et al. Imipramine in patients with chest pain despite normal coronary angiograms. N Engl J Med. 1994;330(20):1411‐1417. 8159194 10.1056/NEJM199405193302003

[181] Cox ID , Hann CM , Kaski JC . Low dose imipramine improves chest pain but not quality of life in patients with angina and normal coronary angiograms. Eur Heart J. 1998;19(2):250‐254. 9519318 10.1053/euhj.1997.0615

[182] Sestito A , Lanza GA , Le Pera D , et al. Spinal cord stimulation normalizes abnormal cortical pain processing in patients with cardiac syndrome X. Pain. 2008;139(1):82‐89. 18440702 10.1016/j.pain.2008.03.015

[183] Sgueglia GA , Sestito A , Spinelli A , et al. Long‐term follow‐up of patients with cardiac syndrome X treated by spinal cord stimulation. Heart (British Cardiac Society). 2007;93(5):591‐597. 17237133 10.1136/hrt.2006.102194 PMC1955539

[184] Kronhaus KD , Lawson WE . Enhanced external counterpulsation is an effective treatment for Syndrome X. Int J Cardiol. 2009;135(2):256‐257. 18590931 10.1016/j.ijcard.2008.03.022

[185] Harris JR , Hale GM , Dasari TW , Schwier NC . Pharmacotherapy of vasospastic angina. J Cardiovasc Pharmacol Ther. 2016;21(5):439‐451. 27081186 10.1177/1074248416640161

[186] Chahine RA , Feldman RL , Giles TD , et al. Randomized placebo‐controlled trial of amlodipine in vasospastic angina. Amlodipine Study 160 Group. J Am Coll Cardiol. 1993;21(6):1365‐1370. 8166777 10.1016/0735-1097(93)90310-w

[187] Suzuki H , Yokoyama K , Akimoto Y , Daida H . Clinical efficacy of benidipine for vasospastic angina pectoris. Arzneimittelforschung. 2007;57(1):20‐25. 17341005 10.1055/s-0031-1296581

[188] Beijk MA , Vlastra WV , Delewi R , et al. Myocardial infarction with non‐obstructive coronary arteries: a focus on vasospastic angina. Neth Heart J. 2019;27(5):237‐245. 30689112 10.1007/s12471-019-1232-7 PMC6470236

[189] Freedman SB , Richmond DR , Kelly DT . Long‐term follow‐up of verapamil and nitrate treatment for coronary artery spasm. Am J Cardiol. 1982;50(4):711‐715. 6812405 10.1016/0002-9149(82)91223-1

[190] Conti CR , Hill JA , Feldman RL , Conti JB , Pepine CJ . Isosorbide dinitrate and nifedipine in variant angina pectoris. Am Heart J. 1985;110(1):251‐256. Pt 2. 3925744 10.1016/0002-8703(85)90495-8

[191] Ong P , Athanasiadis A , Sechtem U . Pharmacotherapy for coronary microvascular dysfunction. Eur Heart J Cardiovasc Pharmacother. 2015;1(1):65‐71. 27533969 10.1093/ehjcvp/pvu020

[192] Chen JW , Lee WL , Hsu NW , et al. Effects of short‐term treatment of nicorandil on exercise‐induced myocardial ischemia and abnormal cardiac autonomic activity in microvascular angina. Am J Cardiol. 1997;80(1):32‐38. 9205016 10.1016/s0002-9149(97)00279-8

[193] Nihei T , Takahashi J , Hao K , et al. Prognostic impacts of Rho‐kinase activity in circulating leucocytes in patients with vasospastic angina. Eur Heart J. 2018;39(11):952‐959. 29165549 10.1093/eurheartj/ehx657

[194] Suda A , Takahashi J , Hao K , et al. Coronary functional abnormalities in patients with angina and nonobstructive coronary artery disease. J Am Coll Cardiol. 2019;74(19):2350‐2360. 31699275 10.1016/j.jacc.2019.08.1056

[195] Asal NJ , Wojciak KA . Effect of cilostazol in treating diabetes‐associated microvascular complications. Endocrine. 2017;56(2):240‐244. 28293857 10.1007/s12020-017-1279-4

[196] Denardo SJ , Wen X , Handberg EM , et al. Effect of phosphodiesterase type 5 inhibition on microvascular coronary dysfunction in women: a Women's Ischemia Syndrome Evaluation (WISE) ancillary study. Clin Cardiol. 2011;34(8):483‐487. 21780138 10.1002/clc.20935 PMC3151010

[197] De Cesare N , Cozzi S , Apostolo A , et al. Facilitation of coronary spasm by propranolol in Prinzmetal's angina: fact or unproven extrapolation? Coron Artery Dis. 1994;5(4):323‐330. 8044344 10.1097/00019501-199404000-00008

[198] Chow CK , Jolly S , Rao‐Melacini P , Fox KAA , Anand SS , Yusuf S . Association of diet, exercise, and smoking modification with risk of early cardiovascular events after acute coronary syndromes. Circulation. 2010;121(6):750‐758. 20124123 10.1161/CIRCULATIONAHA.109.891523

[199] Booth JN , Levitan EB , Brown TM , Farkouh ME , Safford MM , Muntner P . Effect of sustaining lifestyle modifications (nonsmoking, weight reduction, physical activity, and mediterranean diet) after healing of myocardial infarction, percutaneous intervention, or coronary bypass (from the REasons for Geographic and Racial Differences in Stroke Study). Am J Cardiol. 2014;113(12):1933‐1940. 24793668 10.1016/j.amjcard.2014.03.033 PMC4348576

[200] Ormel J , Von Korff M , Burger H , et al. Mental disorders among persons with heart disease—results from World Mental Health surveys. Gen Hosp Psychiatry. 2007;29(4):325‐334. 17591509 10.1016/j.genhosppsych.2007.03.009 PMC2048744

[201] Baigent C , Keech A , Kearney PM , et al. Efficacy and safety of cholesterol‐lowering treatment: prospective meta‐analysis of data from 90,056 participants in 14 randomised trials of statins. Lancet. 2005;366(9493):1267‐1278. 16214597 10.1016/S0140-6736(05)67394-1

[202] Baigent C , Blackwell L , Emberson J , et al. Efficacy and safety of more intensive lowering of LDL cholesterol: a meta‐analysis of data from 170,000 participants in 26 randomised trials. Lancet. 2010;376(9753):1670‐1681. 21067804 10.1016/S0140-6736(10)61350-5 PMC2988224

[203] Pfeffer MA , Braunwald E , Moyé LA , et al. Effect of captopril on mortality and morbidity in patients with left ventricular dysfunction after myocardial infarction. Results of the survival and ventricular enlargement trial. The SAVE Investigators. N Engl J Med. 1992;327(10):669‐677. 1386652 10.1056/NEJM199209033271001

[204] Flather MD , Yusuf S , Køber L , et al. Long‐term ACE‐inhibitor therapy in patients with heart failure or left‐ventricular dysfunction: a systematic overview of data from individual patients. ACE‐Inhibitor Myocardial Infarction Collaborative Group. Lancet. 2000;355(9215):1575‐1581. 10821360 10.1016/s0140-6736(00)02212-1

[205] Bangalore S , Makani H , Radford M , et al. Clinical outcomes with β‐blockers for myocardial infarction: a meta‐analysis of randomized trials. Am J Med. 2014;127(10):939‐953. 24927909 10.1016/j.amjmed.2014.05.032

[206] Hong J , Barry AR . Long‐term beta‐blocker therapy after myocardial infarction in the reperfusion era: a systematic review. Pharmacotherapy. 2018;38(5):546‐554. 29601115 10.1002/phar.2110

[207] Choi WG , Kim GC , Lee CH , Kim HY , Kim DW . The effect of antiplatelet drug on coronary endothelial and microvascular function: comparison with ticagrelor and clopidogrel. Korean J Intern Med. 2021;36(2):352‐361. 32564571 10.3904/kjim.2019.293 PMC7969081

[208] Neumann FJ , Sousa‐Uva M , Ahlsson A , et al. 2018. Eur Heart J. 2019;40(2):87‐165. 30615155 10.1093/eurheartj/ehy855

[209] Agewall S , Beltrame JF , Reynolds HR , et al. ESC working group position paper on myocardial infarction with non‐obstructive coronary arteries. Eur Heart J. 2017;38(3):143‐153. 28158518 10.1093/eurheartj/ehw149

[210] Lindahl B , Baron T , Erlinge D , et al. Medical therapy for secondary prevention and long‐term outcome in patients with myocardial infarction with nonobstructive coronary artery disease. Circulation. 2017;135(16):1481‐1489. 28179398 10.1161/CIRCULATIONAHA.116.026336

[211] Choo EH , Chang K , Lee KY , et al. Prognosis and predictors of mortality in patients suffering myocardial infarction with non‐obstructive coronary arteries. J Am Heart Assoc. 2019;8(14):e011990. 31284804 10.1161/JAHA.119.011990 PMC6662150

[212] Paolisso P , Bergamaschi L , Saturi G , et al. Secondary prevention medical therapy and outcomes in patients with myocardial infarction with non‐obstructive coronary artery disease. Front Pharmacol. 2019;10:1606. 32082147 10.3389/fphar.2019.01606 PMC7005107

[213] Ishii M , Kaikita K , Sato K , et al. Impact of aspirin on the prognosis in patients with coronary spasm without significant atherosclerotic stenosis. Int J Cardiol. 2016;220:328‐332. 27390950 10.1016/j.ijcard.2016.06.157

[214] Montone RA , Cosentino N , Graziani F , et al. Precision medicine versus standard of care for patients with myocardial infarction with non‐obstructive coronary arteries (MINOCA): rationale and design of the multicentre, randomised PROMISE trial. EuroIntervention. 2022;18(11):e933‐e939. 35734824 10.4244/EIJ-D-22-00178 PMC9743237

[215] Ibanez B , James S , Agewall S , et al. 2017. ESC Guidelines for the management of acute myocardial infarction in patients presenting with ST‐segment elevation: The Task Force for the management of acute myocardial infarction in patients presenting with ST‐segment elevation of the European Society of Cardiology (ESC). Eur Heart J. 2018;39(2):119‐177. 28886621 10.1093/eurheartj/ehx393

[216] van Leeuwen MAH , van der Hoeven NW , Janssens GN , et al. Evaluation of microvascular injury in revascularized patients with ST‐Segment‐Elevation myocardial infarction treated with ticagrelor versus prasugrel. Circulation. 2019;139(5):636‐646. 30586720 10.1161/CIRCULATIONAHA.118.035931

[217] Zeymer U , Hohlfeld T , Vom Dahl J , et al. Prospective, randomised trial of the time dependent antiplatelet effects of 500 and 250 mg acetylsalicylic acid i. v. and 300 mg p. o. in ACS (ACUTE). Thromb Haemost. 2017;117(3):625‐635. 28102427 10.1160/TH16-08-0650

[218] Wiviott SD , Braunwald E , McCabe CH , et al. Prasugrel versus clopidogrel in patients with acute coronary syndromes. N Engl J Med. 2007;357(20):2001‐2015. 17982182 10.1056/NEJMoa0706482

[219] Paraskevaidis IA , Iliodromitis EK , Ikonomidis I , et al. The effect of acute administration of statins on coronary microcirculation during the pre‐revascularization period in patients with myocardial infraction. Atherosclerosis. 2012;223(1):184‐189. 22648087 10.1016/j.atherosclerosis.2012.04.002

[220] Post S , Post MC , van den Branden BJ , et al. Early statin treatment prior to primary PCI for acute myocardial infarction: rEPERATOR, a randomized placebo‐controlled pilot trial. Catheter Cardiovasc Interv. 2012;80(5):756‐765. 22419603 10.1002/ccd.23449

[221] Ma Q , Ma Y , Wang X , et al. Intracoronary compared with intravenous bolus tirofiban on the microvascular obstruction in patients with STEMI undergoing PCI: a cardiac MR study. Int J Cardiovasc Imaging. 2020;36(6):1121‐1132. 32078096 10.1007/s10554-020-01800-0

[222] Sezer M , Oflaz H , Gören T , et al. Intracoronary streptokinase after primary percutaneous coronary intervention. N Engl J Med. 2007;356(18):1823‐1834. 17476008 10.1056/NEJMoa054374

[223] McCartney PJ , Eteiba H , Maznyczka AM , et al. Effect of low‐dose intracoronary alteplase during primary percutaneous coronary intervention on microvascular obstruction in patients with acute myocardial infarction: a randomized clinical trial. JAMA. 2019;321(1):56‐68. 30620371 10.1001/jama.2018.19802 PMC6583564

[224] Niccoli G , Rigattieri S , De Vita MR , et al. Open‐label, randomized, placebo‐controlled evaluation of intracoronary adenosine or nitroprusside after thrombus aspiration during primary percutaneous coronary intervention for the prevention of microvascular obstruction in acute myocardial infarction: the REOPEN‐AMI study (Intracoronary Nitroprusside Versus Adenosine in Acute Myocardial Infarction). JACC Cardiovasc Interv. 2013;6(6):580‐589. 23683738 10.1016/j.jcin.2013.02.009

[225] Zhou J , Xu J , Cheng A , Li P , Chen B , Sun S . Effect of nicorandil treatment adjunctive to percutaneous coronary intervention in patients with acute myocardial infarction: a systematic review and meta‐analysis. J Int Med Res. 2020;48(11):300060520967856. 33249959 10.1177/0300060520967856 PMC7708727

[226] Qian G , Zhang Y , Dong W , et al. Effects of nicorandil administration on infarct size in patients with ST‐Segment‐Elevation myocardial infarction undergoing primary percutaneous coronary intervention: the CHANGE trial. J Am Heart Assoc. 2022;11(18):e026232. 36073634 10.1161/JAHA.122.026232 PMC9683654

[227] Zhao S , Qi G , Tian W , Chen L , Sun Y . Effect of intracoronary nitroprusside in preventing no reflow phenomenon during primary percutaneous coronary intervention: a meta‐analysis. J Interv Cardiol. 2014;27(4):356‐364. 25041036 10.1111/joic.12133

[228] Su Q , Li L , Naing KA , Sun Y . Safety and effectiveness of nitroprusside in preventing no‐reflow during percutaneous coronary intervention: a systematic review. Cell Biochem Biophys. 2014;68(1):201‐206. 23749494 10.1007/s12013-013-9690-9

[229] Huang D , Qian J , Ge L , et al. REstoration of COronary flow in patients with no‐reflow after primary coronary interVEntion of acute myocaRdial infarction (RECOVER). Am Heart J. 2012;164(3):394‐401. 22980307 10.1016/j.ahj.2012.06.015

[230] Jolly SS , James S , Džavík V , et al. Thrombus aspiration in ST‐Segment‐Elevation myocardial infarction: an individual patient meta‐analysis: thrombectomy trialists collaboration. Circulation. 2017;135(2):143‐152. 27941066 10.1161/CIRCULATIONAHA.116.025371

[231] Mauri L , Cox D , Hermiller J , et al. The PROXIMAL trial: proximal protection during saphenous vein graft intervention using the proxis embolic protection system: a randomized, prospective, multicenter clinical trial. J Am Coll Cardiol. 2007;50(15):1442‐1449. 17919563 10.1016/j.jacc.2007.06.039

[232] Shibata N , Takagi K , Morishima I , et al. The impact of the excimer laser on myocardial salvage in ST‐elevation acute myocardial infarction via nuclear scintigraphy. Int J Cardiovasc Imaging. 2020;36(1):161‐170. 31451993 10.1007/s10554-019-01690-x

[233] Carrick D , Oldroyd KG , McEntegart M , et al. A randomized trial of deferred stenting versus immediate stenting to prevent no‐ or slow‐reflow in acute ST‐segment elevation myocardial infarction (DEFER‐STEMI). J Am Coll Cardiol. 2014;63(20):2088‐2098. 24583294 10.1016/j.jacc.2014.02.530 PMC4029071

[234] Hausenloy DJ , Chilian W , Crea F , et al. The coronary circulation in acute myocardial ischaemia/reperfusion injury: a target for cardioprotection. Cardiovasc Res. 2019;115(7):1143‐1155. 30428011 10.1093/cvr/cvy286 PMC6529918

[235] Pizarro G , Fernández‐Friera L , Fuster V , et al. Long‐term benefit of early pre‐reperfusion metoprolol administration in patients with acute myocardial infarction: results from the METOCARD‐CNIC trial (effect of metoprolol in cardioprotection during an acute myocardial infarction). J Am Coll Cardiol. 2014;63(22):2356‐2362. 24694530 10.1016/j.jacc.2014.03.014

[236] García‐Prieto J , Villena‐Gutiérrez R , Gómez M , et al. Neutrophil stunning by metoprolol reduces infarct size. Nat Commun. 2017;8:14780. 28416795 10.1038/ncomms14780 PMC5399300

[237] Roolvink V , Ibáñez B , Ottervanger JP , et al. Early intravenous beta‐blockers in patients with ST‐Segment elevation myocardial infarction before primary percutaneous coronary intervention. J Am Coll Cardiol. 2016;67(23):2705‐2715. 27050189 10.1016/j.jacc.2016.03.522

[238] García‐Ruiz JM , Fernández‐Jiménez R , García‐Alvarez A , et al. Impact of the timing of metoprolol administration during STEMI on infarct size and ventricular function. J Am Coll Cardiol. 2016;67(18):2093‐2104. 27052688 10.1016/j.jacc.2016.02.050

[239] Kim J‐S , Kim J , Choi D , et al. Efficacy of high‐dose atorvastatin loading before primary percutaneous coronary intervention in ST‐segment elevation myocardial infarction: the STATIN STEMI trial. JACC Cardiovasc Interv. 2010;3(3):332‐339. 20298994 10.1016/j.jcin.2009.11.021

[240] Berwanger O , Santucci EV , de Barros E , Silva PGM , et al. Effect of loading dose of atorvastatin prior to planned percutaneous coronary intervention on major adverse cardiovascular events in acute coronary syndrome: the SECURE‐PCI randomized clinical trial. JAMA. 2018;319(13):1331‐1340. 29525821 10.1001/jama.2018.2444 PMC5876881

[241] Mahaffey KW , Puma JA , Barbagelata NA , et al. Adenosine as an adjunct to thrombolytic therapy for acute myocardial infarction: results of a multicenter, randomized, placebo‐controlled trial: the Acute Myocardial Infarction STudy of ADenosine (AMISTAD) trial. J Am Coll Cardiol. 1999;34(6):1711‐1720. 10577561 10.1016/s0735-1097(99)00418-0

[242] Ross AM , Gibbons RJ , Stone GW , Kloner RA , Alexander RW . A randomized, double‐blinded, placebo‐controlled multicenter trial of adenosine as an adjunct to reperfusion in the treatment of acute myocardial infarction (AMISTAD‐II). J Am Coll Cardiol. 2005;45(11):1775‐1780. 15936605 10.1016/j.jacc.2005.02.061

[243] Zhang H , Tian NL , Hu ZY , et al. Three hours continuous injection of adenosine improved left ventricular function and infarct size in patients with ST‐segment elevation myocardial infarction. Chin Med J (Engl). 2012;125(10):1713‐1719. 22800889

[244] Chen W , Spitzl A , Mathes D , et al. Endothelial actions of ANP enhance myocardial inflammatory infiltration in the early phase after acute infarction. Circ Res. 2016;119(2):237‐248. 27142162 10.1161/CIRCRESAHA.115.307196

[245] Kitakaze M , Asakura M , Kim J , et al. Human atrial natriuretic peptide and nicorandil as adjuncts to reperfusion treatment for acute myocardial infarction (J‐WIND): two randomised trials. Lancet. 2007;370(9597):1483‐1493. 17964349 10.1016/S0140-6736(07)61634-1

[246] Van't Hof AWJ , Ten Berg J , Heestermans T , et al. Prehospital initiation of tirofiban in patients with ST‐elevation myocardial infarction undergoing primary angioplasty (On‐TIME 2): a multicentre, double‐blind, randomised controlled trial. Lancet. 2008;372(9638):537‐546. 18707985 10.1016/S0140-6736(08)61235-0

[247] Stone GW , Maehara A , Witzenbichler B , et al. Intracoronary abciximab and aspiration thrombectomy in patients with large anterior myocardial infarction: the INFUSE‐AMI randomized trial. JAMA. 2012;307(17):1817‐1826. 22447888 10.1001/jama.2012.421

[248] Yang HT , Xiu WJ , Zheng YY , Ma YT , Xie X . Effects of erythropoietin on the clinical outcomes of patients with acute ST segment elevation myocardial infarction after percutaneous coronary intervention: a meta‐analysis. Int J Clin Pharmacol Ther. 2018;56(6):277‐279. 29628023 10.5414/CP203225

[249] Chan W , Taylor AJ , Ellims AH , et al. Effect of iron chelation on myocardial infarct size and oxidative stress in ST‐elevation‐myocardial infarction. Circ Cardiovasc Interv. 2012;5(2):270‐278. 22496085 10.1161/CIRCINTERVENTIONS.111.966226

[250] Sloth AD , Schmidt MR , Munk K , et al. Improved long‐term clinical outcomes in patients with ST‐elevation myocardial infarction undergoing remote ischaemic conditioning as an adjunct to primary percutaneous coronary intervention. Eur Heart J. 2014;35(3):168‐175. 24031025 10.1093/eurheartj/eht369

[251] White SK , Frohlich GM , Sado DM , et al. Remote ischemic conditioning reduces myocardial infarct size and edema in patients with ST‐segment elevation myocardial infarction. JACC Cardiovasc Interv. 2015;8(1):178‐188. Pt B. 25240548 10.1016/j.jcin.2014.05.015

[252] Hausenloy DJ , Kharbanda RK , Møller UK , et al. Effect of remote ischaemic conditioning on clinical outcomes in patients with acute myocardial infarction (CONDI‐2/ERIC‐PPCI): a single‐blind randomised controlled trial. Lancet. 2019;394(10207):1415‐1424. 31500849 10.1016/S0140-6736(19)32039-2 PMC6891239

[253] Francis R , Chong J , Ramlall M , et al. Effect of remote ischaemic conditioning on infarct size and remodelling in ST‐segment elevation myocardial infarction patients: the CONDI‐2/ERIC‐PPCI CMR substudy. Basic Res Cardiol. 2021;116(1):59. 34648075 10.1007/s00395-021-00896-2 PMC8516772

[254] De Maria GL , Alkhalil M , Borlotti A , et al. Index of microcirculatory resistance‐guided therapy with pressure‐controlled intermittent coronary sinus occlusion improves coronary microvascular function and reduces infarct size in patients with ST‐elevation myocardial infarction: the Oxford Acute Myocardial Infarction—Pressure‐controlled Intermittent Coronary Sinus Occlusion study (OxAMI‐PICSO study). EuroIntervention. 2018;14(3):e352‐e359. 29792403 10.4244/EIJ-D-18-00378

[255] Parodi O , Neglia D , Sambuceti G , Marabotti C , Palombo C , Donato L . Regional myocardial blood flow and coronary reserve in hypertensive patients. The effect of therapy. Drugs. 1992;44(1):48‐55. Suppl. 1283584 10.2165/00003495-199200441-00009

[256] Parodi O , Neglia D , Palombo C , et al. Comparative effects of enalapril and verapamil on myocardial blood flow in systemic hypertension. Circulation. 1997;96(3):864‐873. 9264494 10.1161/01.cir.96.3.864

[257] Motz W , Strauer BE . Improvement of coronary flow reserve after long‐term therapy with enalapril. Hypertension. 1996;27(5):1031‐1038. 8621193 10.1161/01.hyp.27.5.1031

[258] Soliman OII , Geleijnse ML , Michels M , et al. Effect of successful alcohol septal ablation on microvascular function in patients with obstructive hypertrophic cardiomyopathy. Am J Cardiol. 2008;101(9):1321‐1327. 18435965 10.1016/j.amjcard.2007.12.032

[259] Jaber WA , Yang EH , Nishimura RA , et al. Immediate improvement in coronary flow reserve after alcohol septal ablation in patients with hypertrophic obstructive cardiomyopathy. Heart (British Cardiac Society). 2009;95(7):564‐569. 18952634 10.1136/hrt.2008.148239

[260] Garcia D , Camici PG , Durand L‐G , et al. Impairment of coronary flow reserve in aortic stenosis. J Appl Physiol (1985). 2009;106(1):113‐121. 18974370 10.1152/japplphysiol.00049.2008

[261] Hildick‐Smith DJ , Shapiro LM . Coronary flow reserve improves after aortic valve replacement for aortic stenosis: an adenosine transthoracic echocardiography study. J Am Coll Cardiol. 2000;36(6):1889‐1896. 11092661 10.1016/s0735-1097(00)00947-5

[262] Konst RE , Guzik TJ , Kaski J‐C , Maas AHEM , Elias‐Smale SE . The pathogenic role of coronary microvascular dysfunction in the setting of other cardiac or systemic conditions. Cardiovasc Res. 2020;116(4):817‐828. 31977015 10.1093/cvr/cvaa009 PMC7526753

[263] Aguiar Rosa S , Rocha Lopes L , Fiarresga A , Ferreira RC , Mota Carmo M . Coronary microvascular dysfunction in hypertrophic cardiomyopathy: pathophysiology, assessment, and clinical impact. Microcirculation. 2021;28(1):e12656. 32896949 10.1111/micc.12656

[264] Galderisi M , D'Errico A . Beta‐blockers and coronary flow reserve: the importance of a vasodilatory action. Drugs. 2008;68(5):579‐590. 18370439 10.2165/00003495-200868050-00002

[265] Petkow Dimitrow P , Krzanowski M , Nizankowski R , Szczeklik A , Dubiel JS . Effect of verapamil on systolic and diastolic coronary blood flow velocity in asymptomatic and mildly symptomatic patients with hypertrophic cardiomyopathy. Heart (British Cardiac Society). 2000;83(3):262‐266. 10677401 10.1136/heart.83.3.262 PMC1729332

[266] Bang CN , Greve AM , Rossebø AB , et al. Antihypertensive treatment with β‐Blockade in patients with asymptomatic aortic stenosis and association with cardiovascular events. J Am Heart Assoc. 2017;6(12):e006709. 29180457 10.1161/JAHA.117.006709 PMC5779004

[267] Mohri M , Tagawa H , Egashira K , Takeshita A . Intracoronary enalaprilat improves metabolic coronary vasodilation in patients with idiopathic dilated cardiomyopathy. J Cardiovasc Pharmacol. 2000;35(2):249‐255. 10672857 10.1097/00005344-200002000-00011

[268] Calişkan M , Ciftçi O , Güllü H , Müderrisoğlu H . The effect of carvedilol therapy on coronary flow reserve in patients with idiopathic dilated cardiomyopathy. Turk Kardiyol Dern Ars. 2008;36(4):247‐252. 18765968

[269] Neglia D , De Maria R , Masi S , et al. Effects of long‐term treatment with carvedilol on myocardial blood flow in idiopathic dilated cardiomyopathy. Heart (British Cardiac Society). 2007;93(7):808‐813. 17237134 10.1136/hrt.2006.095208 PMC1994449

[270] Erdogan D , Gullu H , Caliskan M , et al. Nebivolol improves coronary flow reserve in patients with idiopathic dilated cardiomyopathy. Heart (British Cardiac Society). 2007;93(3):319‐324. 17065184 10.1136/hrt.2006.091751 PMC1861460

[271] Skalidis EI , Hamilos MI , Chlouverakis G , Kochiadakis GE , Parthenakis FI , Vardas PE . Acute effect of esmolol intravenously on coronary microcirculation in patients with idiopathic dilated cardiomyopathy. Am J Cardiol. 2007;100(8):1299‐1302. 17920374 10.1016/j.amjcard.2007.05.055

[272] Erdogan D , Tayyar S , Uysal BA , et al. Effects of allopurinol on coronary microvascular and left ventricular function in patients with idiopathic dilated cardiomyopathy. Can J Cardiol. 2012;28(6):721‐727. 22717250 10.1016/j.cjca.2012.04.005

